# Palmoplantare Pustulose: Entstehung, Differentialdiagnose und Therapie

**DOI:** 10.1111/ddg.70238

**Published:** 2026-04-08

**Authors:** Rotraut Mössner, Tanja Fetter, Robert Sabat, Ulrich Mrowietz, Neda Cramer, Dagmar Wilsmann‐Theis

**Affiliations:** ^1^ Klinik für Dermatologie Venerologie und Allergologie Universitätsmedizin Göttingen Göttingen Deutschland; ^2^ Zentrum für Hauterkrankungen Klinik für Dermatologie und Allergologie Universitätsklinikum Bonn Bonn Deutschland; ^3^ Translationale Entzündungsforschung der Haut Klinik für Dermatologie Venerologie und Allergologie Charité – Universitätsmedizin Berlin Corporate member von Freie Universität Berlin und Humboldt‐Universität zu Berlin Berlin Deutschland; ^4^ Zentrum für entzündliche Hauterkrankungen Klinik für Dermatologie Venereologie und Allergologie Universitätsklinikum Schleswig‐Holstein, Campus Kiel

**Keywords:** PAO, palmoplantare Pustulose, Pustulosis palmoplantaris, Psoriasis pustulosa palmoplantaris, SAPHO, Therapie, PAO, palmoplantar Pustulosis, Pustulosis palmoplantar, Palmoplantar pustular psoriasis, SAPHO, Therapy

## Abstract

Die palmoplantare Pustulose (PPP) ist eine chronisch entzündliche, häufig schmerzhafte Erkrankung mit sterilen Pusteln an Handflächen und Fußsohlen, die die Lebensqualität stark einschränkt. Frauen sind häufiger betroffen als Männer, und Rauchen ist ein bedeutender Provokationsfaktor. Unter Therapie mit Biologika, vor allem TNF‐Antagonisten, kann eine sogenannte paradoxe PPP auftreten. Die PPP ist mit der Psoriasis vulgaris assoziiert und kann mit osteoartikulärer Beteiligung einhergehen. Pathogenetisch beginnt die PPP wahrscheinlich um das Acrosyringium. Die Pusteln bestehen überwiegend aus neutrophilen Granulozyten, die durch von aktivierten Keratinozyten sezernierte chemotaktische Faktoren angelockt werden. Die Entzündung wird durch einen sich selbst verstärkenden Mechanismus aufrechterhalten, an dem Interleukin (IL)‐17, IL‐19 und weitere Mediatoren beteiligt sind.

Bei der PPP werden topische Therapien, UV‐Photo‐Therapien, vor allem als topische PUVA‐Therapie (Psoralen plus UVA‐Strahlung), und Systemtherapien eingesetzt. Die systemischen Therapien umfassen konventionelle Medikamente wie Acitretin, Methotrexat, Fumarsäureester und Ciclosporin, neuere *small molecules* wie Apremilast und Januskinase‐Inhibitoren sowie Biologika. Konventionelle Systemtherapien sind bei der PPP oft nicht ausreichend wirksam und nebenwirkungsbehaftet. Aktuell besitzt von den Systemtherapien nur Acitretin eine Zulassung für die PPP. In den letzten Jahren konnten in placebokontrollierten Studien signifikante Effekte von Apremilast, Brodalumab, Guselkumab und Risankizumab auf die PPP gezeigt werden, und weitere Studien mit topischen und systemischen Januskinase‐Inhibitoren sowie IL17A/F‐ Inhibitoren werden durchgeführt.

## EINLEITUNG

Neben der Psoriasis vulgaris (PV) gibt es auch die sogenannte pustulöse Psoriasis. Hierzu gehören historisch betrachtet generalisierte Formen wie die generalisierte pustulöse Psoriasis (GPP) mit ihren Sonderformen, sowie lokalisierte Formen, die überwiegend an Händen und Füßen auftreten. Hierzu gehören die palmoplantare Pustulose (PPP) als häufigste Form, die 1930 von Barber als pustulöse Form der Psoriasis beschrieben wurde,^1^ das 1934 beschriebene pustulöse Bakterid (Andrews) als akute PPP,^1^, ^2^ sowie die Acrodermatitis continua suppurativa Hallopeau, die das Nagelorgan betrifft. Die Einstufung dieser pustulösen Erkrankungen als Sonderformen der Psoriasis war schon früh umstritten. Ursprünglich fiel unter anderem das im Vergleich zur PV schlechte Therapieansprechen der PPP auf.^3^ Die Betrachtung der pustulösen Psoriasisformen als eigene Entitäten wurde später durch genetische Unterschiede erhärtet.^3^, ^4^ So wird auch anstatt der früheren Bezeichnung Psoriasis pustulosa palmoplantaris zunehmend die Bezeichnung Pustulosis palmoplantaris oder palmoplantare Pustulose  verwendet.^5^ Das *European Rare and Severe Expert Network* (ERASPEN) definierte die PPP im Jahr 2017 als primäre, persistierende (> 3 Monate) sterile, makroskopisch sichtbare Pusteln der Handflächen und/oder Fußsohlen.^6^

In diesem Artikel werden pathophysiologische Grundlagen und aktuelle Therapien der PPP dargestellt.

Die palmoplantare Pustulose ist eine chronische, rezidivierende Hauterkrankung, die durch sterile Pusteln auf erythematösen Grund plantar und/oder palmar charakterisiert ist und mit Schmerz und Juckreiz einhergeht.

## EPIDEMIOLOGIE UND KLINISCHES BILD

**ABBILDUNG 1:**
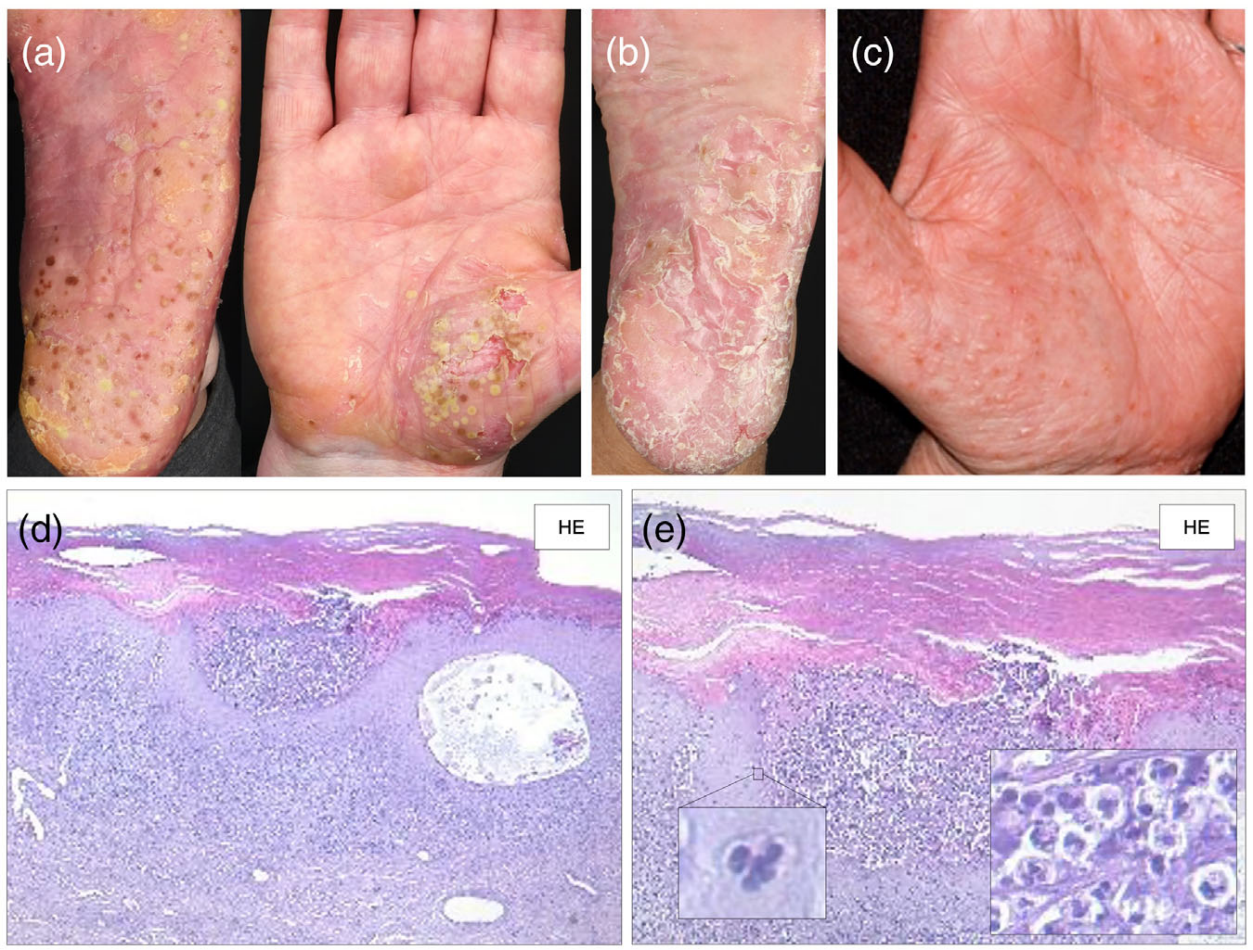
Klinische Varianten und histologische Merkmale der palmoplantaren Pustulose (PPP). Prädilektionsorte der PPP sind plantar vor allem der Hohlfuß, aber auch die Fußränder und Fersen, und palmar die Thenar‐ (a) und Hypothenarregionen. Die sterilen Pusteln können konfluieren und sich im Verlauf als braune Maculae (a) und/oder unter Bildung hyperkeratotischer Plaques, die der Plaque‐Psoriasis ähneln (b), auflösen. (a) Pustulöse Variante, (b) Hyperkeratotische Variante, (c) Dyshidrosiforme Variante, (d) und (e) Histologie einer PPP. Palmoplantaren Pustulose mit Kogojschen Makropusteln, bestehend aus dichten intraepidermalen Ansammlungen neutrophiler Granulozyten. Teilweise finden sich Munro‐Mikroabszesse im Stratum corneum. Die Dermis zeigt ein gemischtzelliges entzündliches Infiltrat mit dilatierten Kapillaren; Eosinophile und Mastzellen können unterhalb der Pusteln vorkommen.

Die PPP ist eine chronische oder chronisch rezidivierende Erkrankung und manifestiert sich klinisch mit Pusteln, oft auf erythematösem und teils schuppigem Grund auf Handflächen und/ oder Fußsohlen.^7^ (Abbildung 1). Plantar sind insbesondere der Hohlfuß, daneben auch die Fußränder und Fersen häufig betroffen; palmar treten die Veränderungen bevorzugt in der Thenar‐ und Hypothenarregion auf. Die sterilen Pusteln können konfluieren und sich im Verlauf als braune Maculae und/oder unter Bildung hyperkeratotischer Plaques, die der Plaque‐Psoriasis ähneln, auflösen.^8^, ^9^ Vesikel, die zu Pusteln werden können, gehören auch zum klinischen Spektrum der PPP,^10^ (Abbildung 1). Bei 30–76 % der Patienten mit PPP bestehen Nagelveränderungen, vor allem Onycholysen bis hin zur Nageldestruktion.^8^, ^11^ Die PPP kann einen jahrzehntelangen Verlauf mit Phasen partieller oder vollständiger Remission, gefolgt von Exazerbationen, zeigen.^12^, ^13^, ^14^ Die Hautläsionen gehen oft mit starken Schmerzen und Jucken einher.^12^

**ABBILDUNG 2:**
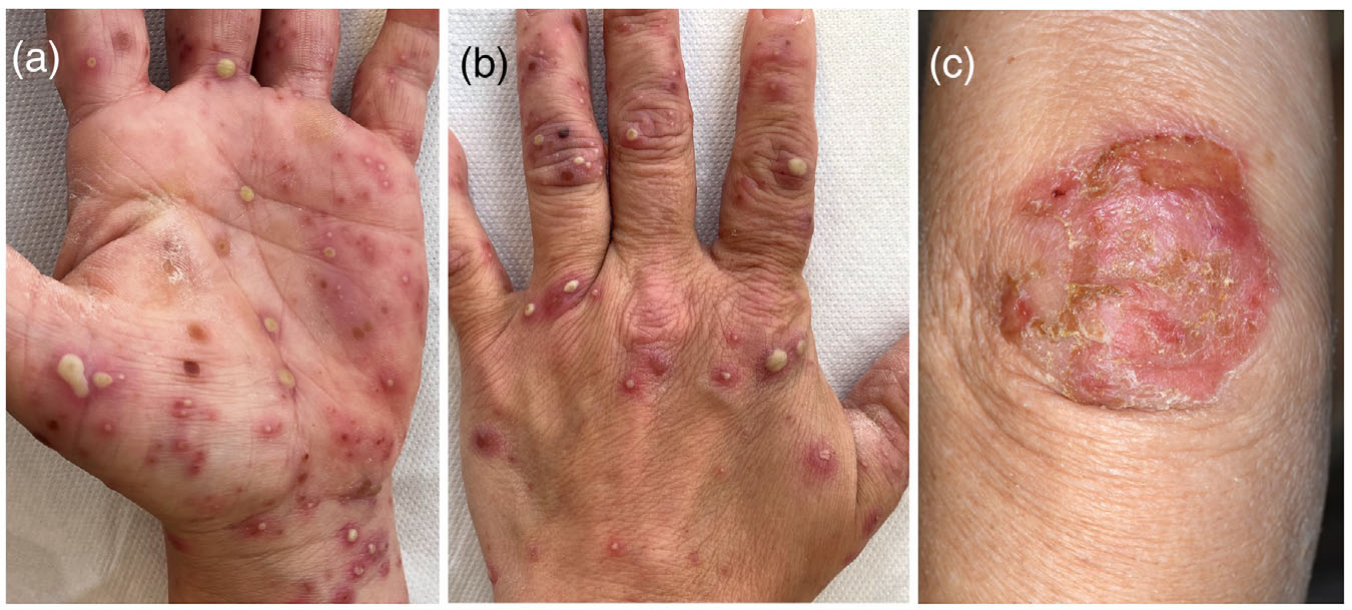
Typisches klinisches Bild einer akuten palmoplantaren Pustulose mit großen Pusteln mit erythematösem Randsaum (a), und t Mitbeteiligung des Handrückens mit einzelnstehenden Pusteln mit erythematösen Randsaum (b), sowie hyperkeratotisch‐erosive Plaques am Ellenbogen (c) auf sonst unbefallener Haut.

Von der (chronischen) PPP wird eine akute PPP unterschieden, die auch pustulöses Bakterid (Andrews) genannt wird,^1^ (Abbildung 2). Ihr geht oft eine bakterielle Infektion, oft durch Streptokokken, vor allem der Tonsillen voraus.^5^ Sie wurde ursprünglich als selbstlimitierende pustulöse Id‐Reaktion angesehen, was aber umstritten bleibt. Klinisch können sich meist einzelnstehende, oft größere Pusteln zwischen 5 und 10 mm Durchmesser mit rotem Randsaum auf sonst unbefallener Haut zeigen. Die Pusteln treten vor allem palmoplantar auf, aber es können auch Pusteln an Hand‐ und Fußrücken sowie vereinzelt am restlichen Integument auftreten (Abbildung 2).^5^, ^15^

Die Prävalenz der PPP ist nicht genau bekannt. Die in Deutschland anhand der IQVIA *Disease Analyzer Database* über den ICD‐10‐CM L40.3 erhobene Prävalenz lag bei 0,065 % und die in den USA (erhoben anhand der *US MerativeTM MarketScan Commercial Database*) bei 0,005 %.^16^ Sie scheint in Japan höher zu liegen (bis 0,12 %) als in westlichen Ländern.^16^, ^17^, ^18^, ^19^ Frauen sind mit einem Anteil zwischen 58 % und 94 % häufiger von PPP betroffen als Männer.^8^ Der Schweregrad der Erkrankung scheint bei Frauen höher zu sein als bei Männern.^17^ In einer Studie aus Deutschland erkrankten die Patienten am häufigsten im Alter zwischen 40 bis 59 Jahren, im Durchschnitt mit 41,6 Jahren.^20^ Die PPP geht mit stark verminderter Lebensqualität einher.^20^ Es besteht eine positive Korrelation zwischen dem Schweregrad der PPP und der Einschränkung der gesundheitsbezogenen Lebensqualität, gemessen mit dem *Palmoplantar Pustulosis Area and Severity Index* (PPPASI) und dem *Dermatology Life Quality Index* (DLQI).^14^, ^15^, ^16^, ^17^, ^18^, ^19^, ^20^

Der Schweregrad der PPP wird sowohl durch patientenorientierte Scores wie DLQI und Evaluation von Schmerz und Jucken (visuelle Analogskala, numerische Bewertungsskala) als auch objektive Befundscores wie dem PPPASI,^21^ oder/und den weniger zeitaufwendigen PPP‐PGA (*Physician Global Assessment*) (Tabelle 1; Tabelle 2) evaluiert.^22^

Die Prävalenz der PPP liegt bei schätzungsweise 0,065 % in Deutschland und postmenopausale Frauen sind hauptsächlich betroffen. Sie wird maßgeblich durch Tabakrauchen provoziert.

**TABELLE 1:** Score zur Einschätzung des objektiven Schweregrades der palmoplantaren Pustulose (PPP): PPP‐*Physician Global Assessment* (PGA)

PGA‐Skala	Kurzbeschreibung	Detaillierte Beschreibung	Klinisches Beispiel
0	erscheinungsfrei	Keine Anzeichen von PPP; keine Schuppung oder Krusten oder Reste von Pusteln	
1	nahezu erscheinungsfrei	Minimale Schuppung und/oder minimales Erythem und/oder leichte Krusten; sehr wenige neue (gelbe) und/oder alte (braune) Pusteln	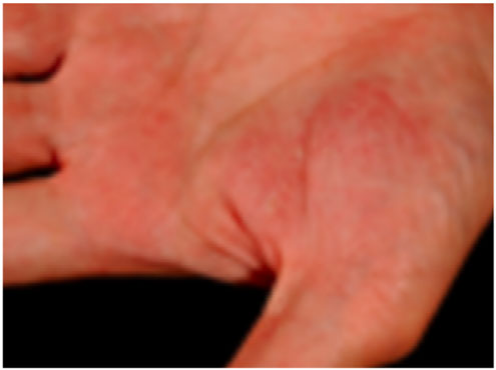
2	leicht	Leichte Schuppung und/oder leichtes Erythem und/oder Krusten; sichtbare neue (gelbe) und/oder alte (braune) Pusteln von begrenzter Anzahl und Ausdehnung	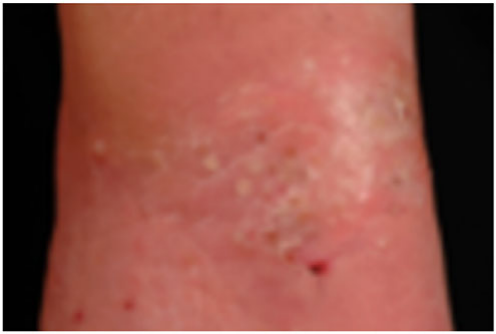
3	mittelschwer	Mittelschwere Schuppung und/oder deutliches Erythem und/oder Krustenbildung; auffällige neue (gelbe) und/oder alte (braune) Pusteln, die den größten Teil des betroffenen Areals bedecken	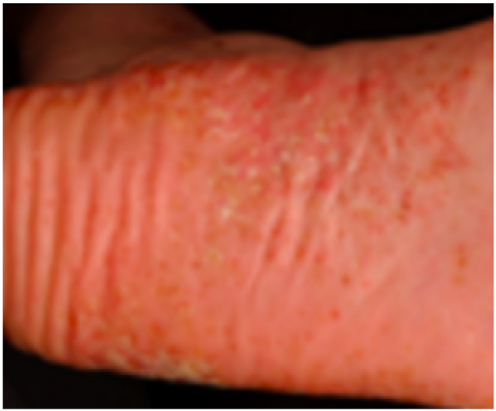
4	schwer	Starke Schuppung und/oder starkes Erythem und/oder Krustenbildung; zahlreiche neue (gelbe) oder alte (braune) Pusteln mit und/oder ohne Konfluenz, die das gesamte Areal von mindestens 2 palmoplantaren Oberflächen bedecken	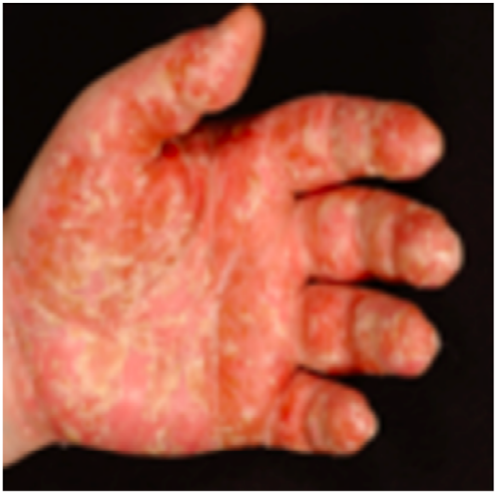

**TABELLE 2:** Score zur Einschätzung des objektiven Schweregrades der palmoplantaren Pustulose (PPP) *Palmoplantar Pustulosis Area and Severity Index* (PPPASI)

Körperregion	Erythem (E)	Pusteln/Vesikeln (P)	Schuppung (Desquamation) (D)	Ausmaß der Beteiligung (A)
**Rechts palmar** **(RP)**	Punktzahl 0–4	Punktzahl 0–4	Punktzahl 0–4	Punktzahl 0–6
**Links palmar** **(LP)**	Punktzahl 0–4	Punktzahl 0–4	Punktzahl 0–4	Punktzahl 0–6
**Rechte Fußsohle** **(RS)**	Punktzahl 0–4	Punktzahl 0–4	Punktzahl 0–4	Punktzahl 0–6
**Linke Fußsohle** **(LS)**	Punktzahl 0–4	Punktzahl 0–4	Punktzahl 0–4	Punktzahl 0–6
**Formel zur** **Berechnung**	**PPPASI** = 0,2 x (ERP + PRP + DRP) x ARP + 0,2 x (ELP + PLP + DLP) x ALP + 0,3 x (ERS + PRS + DRS) x ARS + 0,3 x (ELS + PLS + DLS) x ALS			
Erörterung Punktzahl gemäß Befall	0 = kein Befall 1 = leicht 2 = moderat 3 = schwer 4 = sehr schwer	0 = kein Befall 1 = leicht 2 = moderat 3 = schwer 4 = sehr schwer	0 = kein Befall 1 = leicht 2 = moderat 3 = schwer 4 = sehr schwer	0 = kein Befall <10 % = 1 10–29 % = 2 30–49 % = 3 50–69 % = 4 70–89 % = 5 90–100 % = 6
Gesamtpunktzahl	72 (Maximum)			

### Provokationsfaktoren

Ein wichtiger Provokationsfaktor der PPP ist das Tabakrauchen. In einem deutschen Kollektiv waren 95 % der Patienten mit PPP Raucher oder Ex‐Raucher.^20^ Es fand sich auch eine schwerere Ausprägung der PPP bei Rauchern im Vergleich zu Ex‐Rauchern und Nichtrauchern,^23^ und in einer kleinen Studie kam es nach Nikotinkarenz zur Besserung der PPP.^24^ Ferner wurden vor allem bei japanischen Patienten Assoziationen der PPP mit Infekten, vor allem Tonsillitiden oder Periodontitis beschrieben.^25^ In einer retrospektiven japanischen Studie lag bei 87 % der 85 eingeschlossenen Patienten eine unbehandelte Zahninfektion vor.^26^ In diesem Kollektiv kam es bei 39 % der Patienten zu einer deutlichen Besserung der PPP nach erfolgreicher zahnärztlicher Behandlung. Bei fünf von sechs Patienten mit einer Tonsillitis kam es nach Tonsillektomie zu einer deutlichen Besserung der PPP.^26^ Überwiegend aus Japan stammen auch weitere, größere Untersuchungen zur therapeutischen Wirkung einer Tonsillektomie bei therapierefraktären PPP‐Patienten. In einer retrospektiven Untersuchung an 138 Patienten in Japan waren 43 % bei ihrer letzten Vorstellung erscheinungsfrei, und in einer Kaplan‐Meier Analyse war die PPP bei 38 % der Patienten innerhalb von zwölf Monaten und bei 66 % nach 24 Monaten abgeheilt.^25^ Interessanterweise waren weder eine Verschlechterung der PPP unter Racheninfekten noch das Vorliegen einer Tonsillenhypertrophie klinische Marker für eine Besserung der PPP nach Tonsillektomie.^25^

Auch mechanische oder chemische Reize, Feuchtarbeit und Hitze können zu einer Verschlechterung der PPP führen.^17^, ^27^, ^28^, ^29^ Da eine PPP oft zu längerfristiger Krankschreibung führt, sollte geprüft werden, ob die PPP durch den Beruf maßgeblich beeinflusst wird und eine Meldung an die zuständige Berufsgenossenschaft erfolgen sollte. Sofern unter Ausübung einer hautgefährdenden Tätigkeit im Sinne der BK Nr. 5101 eine Arbeitskongruenz der Hauterscheinungen deutlich wird, sollte dies dem zuständigen Unfallversicherungsträger angezeigt werden, in Deutschland mittels Hautarztbericht (Einleitung Hautarztverfahren, Formular F6050). Häufiger ist es bei der PPP jedoch so, dass die beruflichen Einwirkungen das Maß einer sogenannten Gelegenheitsursache nicht überschreiten.^29^

Psychologischer Stress wird von Patienten mit PPP als Auslöser oder Ursache für eine Verschlimmerung angegeben.^20^ Kontaktallergien werden als Provokationsfaktoren einer PPP kontrovers diskutiert. Eine systematische Übersichtsarbeit ergab bei 22,7 % der PPP‐Patienten epikutane Sensibilisierungen, vor allem auf Metalle.^30^ In dieser Übersichtsarbeit wurde nach Entfernung von Zahnfüllungen oder Zahnprothesen bei Patienten mit einem positiven Epikutantest eine Besserung der PPP beobachtet,^30^ wobei eine solche Besserung in einer Studie eher auf die gleichzeitige Behandlung von Zahninfektionen zurückgeführt wurde,^31^ und in einer weiteren Studie nach Entfernung von Metallen im Mundraum sechs von neun Patienten keine Besserung zeigten.^26^

Die paradoxe PPP kann als  Nebenwirkung vor allem unter TNF‐Blockern auftreten, insbesondere bei Patienten mit chronisch entzündlichen Darmerkrankungen.

### Paradoxe PPP

Unter bestimmten Biologika‐Therapien wurden psoriatische Hautveränderungen als sogenannte paradoxe Reaktionen beobachtet. Paradox wird hier in dem Sinne verwendet, dass die induzierte Nebenwirkung einer Erkrankung ähnelt, zu deren Therapie das auslösende Medikament eingesetzt wird.^5^ Bei den paradoxen psoriasiformen Reaktionen kann es sich um eine Verschlechterung einer bestehenden Psoriasis handeln, um eine Änderung des klinischen Bildes wie zum Beispiel dem erstmaligen Auftreten einer pustulösen Variante, oder aber um eine Erstmanifestation psoriasiformer Hautveränderungen.^5^ 14 % bis 75 % der paradoxen psoriasiformen Reaktionen sind pustulös. Dabei sind vor allem Palmae und Plantae betroffen, was auch als paradoxe PPP bezeichnet wird, während ein generalisierter Befall nur bei etwa 22 % der Patienten mit einer pustulösen Variante beobachtet wurde.^32^ Am häufigsten tritt die paradoxe PPP bei Therapie mit Tumor‐Nekrose‐Faktor (TNF)‐α Blockern auf.^33^ Selten wurde die paradoxe PPP auch bei IL‐17‐ oder IL‐17RA‐Antagonisten beschrieben.^34^ Die Latenz nach Therapiebeginn mit einem TNF‐Blocker beträgt durchschnittlich 10,5 Monate, und lag zwischen einigen Tagen und 80 Monaten.^35^ Paradoxe durch TNF‐Blocker induzierte psoriasiforme Reaktionen wurden in allen zugelassenen Indikationen beobachtet, wobei sie besonders häufig bei Patienten mit entzündlichen Darmerkrankungen berichtet wurden, während sie bei Patienten mit rheumatoider Arthritis seltener waren.^5^ Als Risikofaktoren für paradoxe psoriasiforme Reaktionen wurden in einer Metaanalyse bei Patienten mit entzündlichen Darmerkrankungen weibliches Geschlecht, Rauchen, jüngeres Alter bei Beginn einer TNF‐Blockertherapie, Patienten mit ilieokolischem Typ des Morbus Crohn und dem TNF‐Blocker‐Präparat (Adalimumab und Certolizumab versus Infliximab) identifiziert.^36^ Es wird angenommen, dass durch Blockade von TNF der Typ I Interferon (IFN)‐Signalweg überaktiviert wird und maßgeblich zur Entstehung der Hautläsionen beiträgt.^37^

## ASSOZIIERTE ERKRANKUNGEN

Die PPP ist häufig mit einer PV assoziiert.^38^ In einem deutschen Kollektiv waren es 25 % der Patienten mit PPP, von denen 50 % angaben, dass die PV im gleichen Jahr wie die PPP aufgetreten sei.^20^ 30,2 % der Patienten mit PPP wiesen in dieser Untersuchung eine positive Familienanamnese für Psoriasis auf.^20^ In Studien zur Genexpression läsionaler Haut von Patienten mit PPP konnten keine signifikanten Unterschiede zwischen den Untergruppen mit oder ohne Psoriasis‐typische Läsionen am restlichen Integument gefunden werden, und es bleibt unklar, ob sich die PPP mit begleitender PV von der PPP ohne PV pathogenetisch unterscheidet.^39^

### Knochen‐ und Gelenkbeteiligung

Die PPP ist mit Knochen‐ und Gelenkbeteiligung assoziiert, die klinisch vielfältig ist. Zentral ist ein Befall mit aseptischer Osteitis mit Osteomyelitis, die sich in der Magnetresonanztomographie als Knochenmarksödem darstellt, und eine Hyperostose mit ossifizierender Periostitis.^40^, ^41^ Ein typisches Beispiel hierfür ist eine Entzündung der vorderen Thoraxwand, die sich klinisch mit einer Schwellung und einer Druckschmerzhaftigkeit der Manubriosternal‐ oder der Costoklavikularregionen äußert, für die Sonozaki in Japan den Begriff pustulöse Arthroosteitis geprägt hat.^42^, ^43^ Die Definition der pustulösen Arthroosteitis, die zwingend die Diagnose einer PPP erfordert, wurde später bezüglich der osteoartikulären Kriterien modifiziert, die inzwischen auch die entzündliche Beteiligung der Wirbelsäule, der Sakroiliakalgelenke oder anderer Knochen umfassen.^44^ Die Prävalenz der pustulösen Arthroosteitis liegt in Japan bei 10–30 % der Patienten mit PPP.^41^ In Deutschland ist der Begriff pustulöse Arthroosteitis weniger bekannt, geläufiger ist das Synovitis‐Acne‐Pustulosis‐Hyperostosis‐Osteitis (SAPHO) Syndrom, das ursprünglich 1987 auf Basis einer nationalen Umfrage in Frankreich definiert wurde.^45^ Es wurden dort vier Hauptkriterien festgelegt, wobei jedes einzelne hinreichend für die Diagnosestellung des SAPHO‐Syndroms ist, sofern keine Ausschlusskriterien (wie eine infektiöse Osteitis) bestehen. Zwei der vier Hauptkriterien stehen im Zusammenhang mit pustulösen Hauterkrankungen, nämlich 1. osteoartikuläre Beteiligung bei Vorliegen einer schweren Akne und 2. osteoartikuläre Beteiligung bei Vorliegen einer PPP. Die beiden weiteren Hauptkritieren können auch ohne Hauterkrankung erfüllt sein und sind 3. sterile Hyperostose (die Hyperostose zählt hier auch als steril bei Nachweis von *Cutibakterium acnes* (früher *Propionobacterium acnes*)) mit oder ohne Dermatose und 4. eine chronisch rekurrierende multifokale Osteomyelitis (CRMO) des axialen oder peripheren Skeletts, mit oder ohne Dermatose. Dabei wird das SAPHO‐Syndrom als Symptomkomplex und nicht als einzelne Krankheitsentität verstanden, wobei auch Modifikationen der Kriterien des SAPHO‐Syndroms vorgeschlagen wurden (siehe Tabelle 3).^40^ Als charakteristische Diagnose in diesem Symptomkomplex gibt es zum Beispiel die Spondarthritis hyperostotica pustulo‐psoriatica, die obligate Trias aus palmoplantarer Pustulose, sternokostoklavikulärer Hyperostose und einer (hyperostotischen) Spondylarthritis. Das SAPHO‐Syndrom umfasst auch die pustulöse Arthroosteitis.^40^, ^41^, ^46^ Als die PPP weniger als eigenständige Entität und eher dem psoriatischen Formenkreis zugehörig eingeordnet wurde, wurden Knochen‐ und Gelenkbeteiligung auch als Psoriasisarthritis (PsA) verstanden.^47^ In einer in Südkorea durchgeführten Untersuchung wurden Patienten mit pustulöser Arthroosteitis und PsA verglichen. Aufgrund von Unterschieden in demographischen Daten und der betroffenen Gelenke werden auch dort die pustulöse Arthroosteitis und die PsA eher als zwei verschiedene Entitäten verstanden.^48^ Weitere Untersuchungen zur Frage, inwieweit die Knochen‐ und Gelenkbeteiligung bei Patienten mit PPP sich in Populationen mit unterschiedlichem genetischen Hintergrund unterscheiden, sind wünschenswert.

**TABELLE 3:** Tabellarische Übersicht über diagnostische Kriterien für die pustulöse Arthroosteitis (Sonozaki‐Syndrom), das SAPHO‐Syndrom und Psoriasisarthritis zur Differenzierung muskuloskelettaler Manifestationen bei der palmoplantaren Pustulose (PPP).

**Pustulöse Arthroosteitis = Sonozaki‐Syndrom**	
**Diagnostische Kriterien^a^ nach Sonozaki et al. (1981)** ^42^ **(adaptiert nach Kishimoto et al. (2022)** ^41^	
1) Diagnostizierte palmoplantare Pustulose	
2) Patienten, die eines der beiden folgenden Kriterien erfüllen: Offensichtliche Schwellung mit Druckschmerz auf beiden Seiten der kostoklavikulären oder manubriosternalen Regionen, mit oder ohne positiven Röntgenbefund Druckschmerz ohne sichtbare Schwellung auf beiden Seiten der kostoklavikulären oder manubriosternalen Regionen, mit positivem Röntgenbefund an der druckschmerzhaften Stelle	
*Diagnosestellung erfolgt bei Vorliegen von:* *Kriterium 1) und 2)*	
*^a^Die diagnostischen Kriterien wurden später modifiziert, siehe zum Beispiel Tsuji et al. (2024)* ^44^ *, Nagel et al. (1993)* ^43^	
**SAPHO‐Syndrom**	
**Diagnostische Kriterien adaptiert nach Kishimoto et al. (2022)** ^41^ **nach Hayem et al. (2004)** ^46^	
Einschlusskriterien: Knochen‐Gelenk‐Beteiligung bei PPP und Psoriasis vulgaris Knochen‐Gelenk‐Beteiligung bei schwerer Akne Isolierte sterile Hyperostose/Osteitis beim Erwachsenen Chronisch rekurrierende multifokale Osteomyelitis beim Kind Knochen‐Gelenk‐Beteiligung bei chronisch‐entzündlichen Darmerkrankungen	
Ausschlusskriterien: Infektiöse Osteitis Tumoröse Erkrankungen des Knochens Nicht‐entzündliche Knochenverdichtungen	
*Diagnosestellung erfolgt bei Vorliegen von:* *Einem Einschlusskriterium, keinem Ausschlusskriterium*	
**Psoriasisarthritis**	
**CASPAR‐Klassifikationskriterien nach Taylor et al. (2006)** ^50^	
*Diagnosestellung erfolgt bei Vorliegen von:* *Entzündlicher Erkrankung der Gelenke, der Wirbelsäule und der Sehnen/Enthesen mit mindestens 3 Punkten aus den folgenden Kategorien*:	
**Nachweis der Psoriasis**	
Bestehende Plaque‐Psoriasis	2 oder
Anamnestisch bekannte Psoriasis (Angaben des Patienten, des Hausarztes, eines Dermatologen oder Rheumatologen)	1 oder
Psoriasis in der Familienanamnese (anamnestisch bekannte Psoriasis bei Verwandten ersten/zweiten Grades nach Angaben des Patienten)	1
**Psoriatrische Nagelveränderungen**	
Tüpfelung, Onycholyse, Hyperkeratose bei der aktuellen körperlichen Untersuchung	1
**Negatives Testergebnis für das Vorhandensein von Rheumafaktor**	
vorzugsweise mittels ELISA oder Nephelometrie, entsprechend dem lokalen Laborreferenzbereich	1
**Daktylitis**	
Bestehende Schwellung des gesamten Fingers	1 oder
Daktylitis in der Vorgeschichte (Diagnose durch Rheumatologen)	1
**Radiologische Zeichen einer juxta‐artikulären Knochenneubildung**	
Undefinierte Verknöcherung in der Nähe der Gelenkränder (aber ohne Osteophytenbildung) auf Röntgenbildern der Hände oder Füße	1

Abk.: CASPAR = Classification Criteria for Psoriatic Arthritis; ELISA = Enzyme‐Linked Immunosorbent Assay; SAPHO = Synovitis, Acne, Pustulosis, Hyperostosis, Osteitis

Die PsA ist eine chronisch entzündliche Erkrankung des Bewegungsapparats mit Arthritis, Enthesitis, Spondylitis und/oder Daktylitis.^49^ Als Diagnosekriterien für die PsA werden die Classification for Psoriatic Arthritis (CASPAR)‐Kriterien (Tabelle 3) verwendet.^50^ Bei einer Psoriasis (zum Beispiel einer Plaque‐Psoriasis) wird diese mit zwei Punkten bewertet, die CASPAR‐Kriterien werden dann bei Vorliegen einer entzündlichen Gelenkerkrankung (nach Ausschluss von Differenzialdiagnosen) in der Regel erfüllt (es reicht hierfür zum Beispiel ein zusätzlich vorliegender negativer Rheumafaktor). Da die PPP zunehmend nicht mehr als Unterform der Psoriasis gesehen wird, können bei Knochen‐ und Gelenkbeteiligung die CASPAR‐Kriterien nur bei Vorliegen weiterer Kriterien, wie einer positiven Familienanamnese für Psoriasis oder psoriatischer Nagelveränderungen, erfüllt werden. Bei Patienten mit PPP lag die Prävalenz einer Gelenkbeteiligung in Deutschland zwischen 16–28 % (damals als PsA erhoben, da die PPP noch als Form der Psoriasis gewertet wurde).^14^, ^20^

Etwa 25 % der Patienten mit PPP leiden ebenfalls an einer Psoriasis vulgaris. Osteoartikulärer Befall besteht in 16–28 % und stellt sich vielfältig dar, wobei eine typische Manifestation die aseptische Osteitis/Hyperostose ist.

### Weitere Assoziationen

Etwa 30 % der Patienten mit PPP wiesen ein metabolisches Syndrom auf. In Untersuchungen in Deutschland lag ein BMI > 30 bei etwa einem Viertel der Patienten mit PPP vor.^14^, ^20^ Hierbei war der BMI insbesondere bei jüngeren Patienten mit PPP mit 27,7 stark erhöht, während in der Altersgruppe ab 65 Jahren bei den Patienten mit PPP mit 24,3 ein niedrigerer BMI als in der Allgemeinbevölkerung vorlag.^20^ Bei der PPP wurden gehäuft Schilddrüsendysfunktionen beobachtet und erhöhte anti‐Thyreoperoxidase Serumspiegel gefunden, und ein Morbus Basedow trat in einer Untersuchung aus Südkorea häufiger bei Patienten mit PPP als bei Patienten mit PV auf.^51^, ^52^ Das Vorliegen weiterer kardiovaskulärer, metabolischer Erkrankungen und Autoimmunerkrankungen bei Patienten mit PPP wurde beschrieben, aber die Stärke der Assoziation zur PPP im Vergleich zur Allgemeinbevölkerung ist bisher nicht geklärt. Es fand sich bei Patienten mit PPP ferner ein gehäuftes Auftreten psychischer Erkrankungen wie Depressionen, bipolare Störungen, Schizophrenie sowie Angst‐ und Essstörungen.^14^, ^53^, ^54^

## GENETIK

Die PPP unterscheidet sich genetisch von der PV, insbesondere fehlt die Assoziation der PPP mit dem *PSORS1*‐Locus (HLA‐Cw*06:02), dem bedeutendsten genetischen Suszeptibilitätslocus der PV.^55^ Es besteht auch keine Assoziation der PPP mit Mutationen im *IL36RN* Gen, das mit der generalisierten pustulösen Psoriasis ‐assoziiert ist. ^56^

In einer genomweiten Metaanalyse wurde eine signifikante Assoziation der PPP zu den FCGR3A/FCGR3B Loci, die Rezeptoren für Immunglobuline kodieren sowie dem CCHCR1 Locus gefunden. Darüber hinaus wurden 13 weitere, wahrscheinliche Suszeptibilitätsregionen für die PPP gefunden.^57^ Insgesamt waren vor allem immunologische Signalwege in der Haut und von zirkulierenden dendritischen Zellen betroffen. Es fanden sich dabei auch Korrelationen mit *single nucleotide polymorphismen* (SNPs) in der IL4/IL13 Genregion, die Th2 Zytokine kodiert. Passend dazu zeigte eine genetische Korrelationsanalyse eine Korrelation mit atopischer Dermatitis, aber nicht mit PV, und eine negative Korrelation mit Colitis ulcerosa.^57^

Die Entzündung der PPP wird durch einen sich selbst verstärkenden Mechanismus aufrechterhalten, an dem IL‐17, IL‐19 und weitere Mediatoren beteiligt sind, die die Neutrophilen‐Chemotaxis weiter stimulieren.

## PATHOGENESE

Die Pathogenese der PPP ist nicht vollständig geklärt. Die Erkrankung scheint um das Acrosyringium, dem intraepidermalen Ausgangskanal der ekkrinen Schweißdrüsen, zu beginnen,^10^, ^58^ wobei die Pusteln fast ausschließlich aus einwandernden neutrophilen Granulozyten bestehen. Diese Neutrophilen werden durch von aktivierten Keratinozyten sezernierte chemotaktische Faktoren (CXCL8, Lipocalin‐2) angelockt, wobei Nikotin als Provokationsfaktor diesen Prozess verstärkt, wie In‐vitro‐Studien nahelegen.^59^, ^60^

Die entstehenden Pusteln beinhalten nahezu ausschließlich eingewanderte neutrophile Granulozyten. Zu einer zentralen Rolle dieser Zellen in der Pathogenese der PPP passen aktuelle Ergebnisse einer genomweiten Assoziationsmetaanalyse, die eine Assoziation von PPP mit den Genloci der Rezeptoren für Immunglobuline (FCGR3A/FCGR3B) findet.^57^ Tatsächlich wird FCGR3B selektiv von neutrophilen Granulozyten und Eosinophilen exprimiert und kann als ein *decoy* Rezeptor Entzündungsprozesse auf unterschiedlicher Weise modulieren. Bei der Einwanderung dieser Zellen in die Epidermis dürften neben dem Chemokinliganden CXCL6^61^, ^62^ weitere chemotaktische Faktoren wie CXCL1, CXCL8 und Lipocalin‐2 eine Rolle spielen.^59^, ^63^ Diese Proteine werden durch aktivierte Keratinozyten sezerniert. Es ist jedoch aktuell unklar, was die Keratinozyten des Acrosyringiums veranlasst, diese chemotaktischen Faktoren zu produzieren. Als Induktoren kommen sowohl Immunmediatoren wie IL‐1β und TNF,^59^ die durch die gewebsständigen Immunzellen, wie dendritische Zellen und Makrophagen produziert werden, als auch Komponenten des Schweißes infrage. Nikotin, als ein Bestandteil des Tabakrauchs, des wichtigsten ätiologischen Faktors der PPP, verstärkt die Chemotaxis neutrophiler Granulozyten.^60^

**ABBILDUNG 3:**
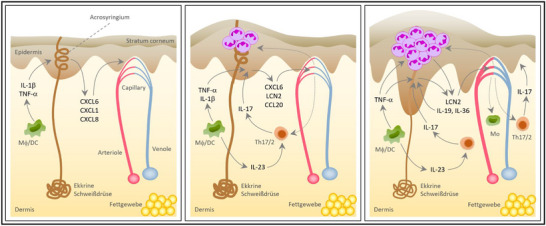
Pathogenese der Palmoplantaren Pustulose (PPP). Die schematische Darstellung veranschaulicht das derzeitige Verständnis der Pathogenese von PPP in drei aufeinanderfolgenden Stadien. (a) Die Erkrankung beginnt um das Acrosyringium. In diesem Bereich der Epidermis werden chemotaktische Faktoren (C‐X‐C *Motif Chemokine Ligand* (CXCL)1, CXCL6, CXCL8), die neutrophile Granulozyten anlocken, von Keratinozyten ausgeschüttet. Mögliche Aktivatoren, die die Keratinozyten dazu veranlassen, sind Bestandteile des Schweißes und die inflammatorischen Zytokine: Interleukin (IL)‐1β und Tumor‐Nekrose‐Faktor (TNF)‐α, die von aktivierten gewebsständigen Immunzellen (Makrophagen, dendritische Zellen; Mf/DC) gebildet werden. (b) Unter Einfluss von IL‐23 werden T‐Helfer (h) 17/Th2‐Zellen aktiviert, die IL‐17 sezernieren. Dieses verstärkt die Produktion chemotaktischer Mediatoren (darunter CXCL6, Lipocalin‐2 [LCN2] und CCL20) durch Keratinozyten. Die neutrophilen Granulozyten akkumulieren intraepidermal zu den für die PPP typischen Kogojschen Mikroabszessen. (c) Die eingewanderten neutrophilen Granulozyten produzieren IL‐19 und LCN2. IL‐19 verstärkt wie IL‐1β und TNF‐α die Wirkung von IL‐17 auf Keratinozyten, und LCN2 fördert die Infiltration und Aktivierung weiterer neutrophiler Granulozyten in der Haut. Der Entzündungszyklus wird chronisch und selbstaufrechthaltend.

In PPP‐Läsionen kann die Produktion von Proteinen aus Keratinozyten, die neutrophile Granulozyten in die Epidermis anlocken, durch Mediatoren wie Interleukin (IL)‐17 und IL‐19 weiter verstärkt werden.^61^, ^64^ IL‐17A wird von eingewanderten T‐Zellen und IL‐19 von neutrophilen Granulozyten produziert.^61^, ^64^, ^65^ Die eingewanderten T‐Zellen sind vor allem in der oberen Dermis zu finden,^28^ und scheinen ein Th17/Th2‐Übergangsphänotyp zu haben.^66^ Diese T‐Zellen exprimieren auch den IL‐23 Rezeptor, und IL‐23 kann ihre Aktivität positiv beeinflussen.^65^, ^66^ Die in die Epidermis eingewanderten neutrophilen Granulozyten produzieren außer IL‐19 auch Lipocalin‐2, welches wiederum im Sinne eines positiven Verstärkers neutrophile Granulozyten in die Epidermis anlockt.^59^, ^67^ Die Wirkung von IL‐17 auf Keratinozyten, die Hauptzielzellen dieses Zytokins, wird durch IL‐1β und TNF verstärkt (Abbildung 3).^59^, ^65^ Die Konzentrationen von sowohl Lipocalin‐2 als auch IL‐19 sind im Blut der Patienten mit PPP erhöht und die Spiegel dieser Mediatoren korrelieren mit der Anzahl der Pusteln.^59^, ^61^

## HISTOLOGIE DER PPP

**ABBILDUNG 4:**
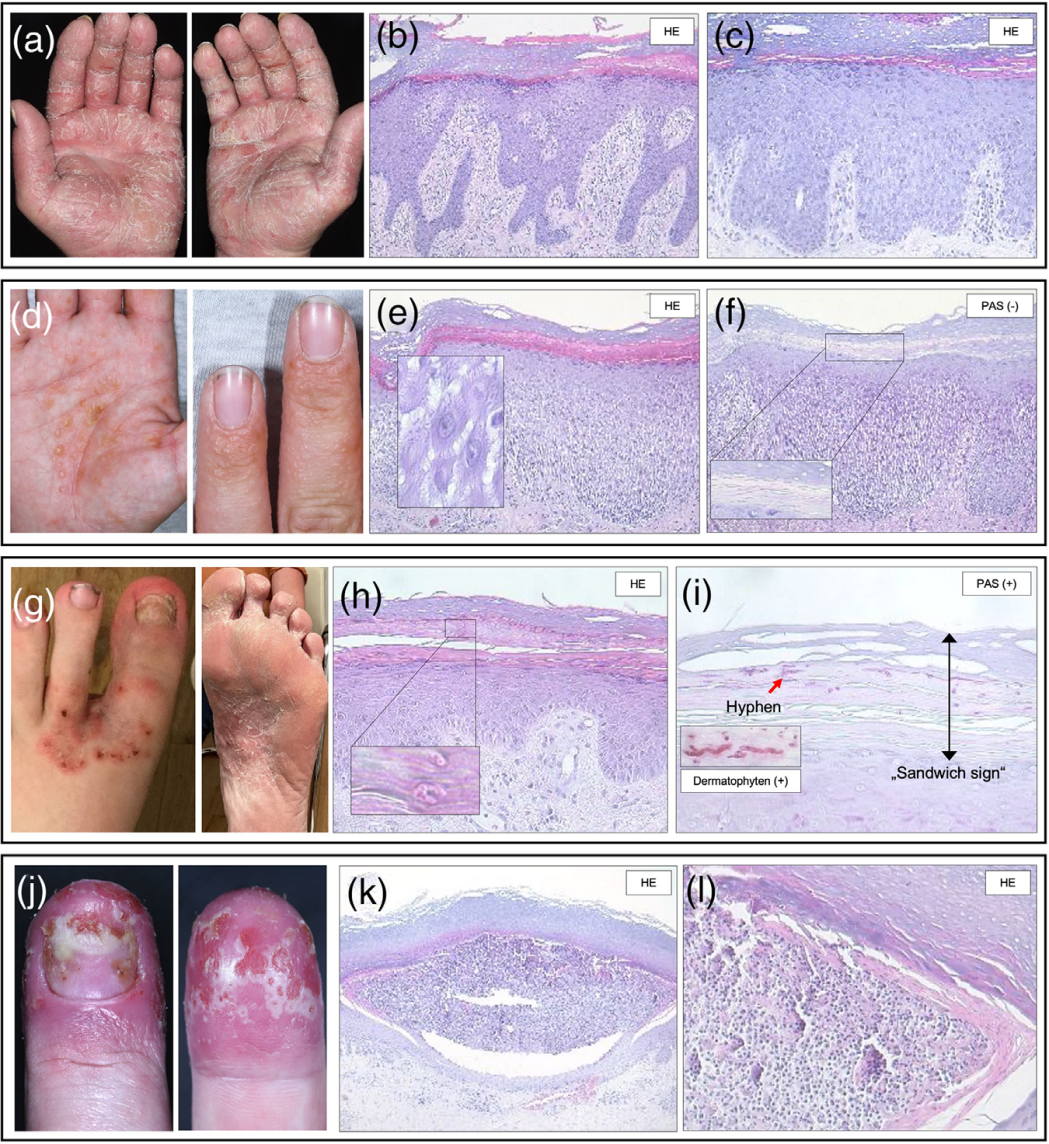
Klinische und histologische Differenzialdiagnosen der palmoplantaren Pustulose (PPP). (a–c) **Chronisches Handekzem** mit Hyperkeratose, Parakeratose, Akanthose und einem perivaskulären lymphozytären Infiltrat, jedoch keinen intraepidermalen Pusteln. (d–f) **Dyshidrosiformes Ekzem** mit ausgeprägter epidermaler Spongiose und Vesikelbildung, aber ohne neutrophile Infiltrate. Die PAS‐Färbung bleibt negativ. (g–i) **Tinea** mit *Periodic Acid Schiff* (PAS)‐positiven Pilzhyphen im Stratum corneum (*Sandwich sign*). Diese Befunde sind entscheidend zur Abgrenzung von PPP, insbesondere bei vesikulären oder pustulösen Läsionen an Handflächen oder Fußsohlen. Eine PAS‐Färbung wird in solchen Fällen stets empfohlen. (j–l) **Acrodermatitis continua suppurativa (Hallopeau)** mit ähnlichen intraepidermalen, spongiformen Pusteln, die mit neutrophilen Granulozyten gefüllt sind, jedoch meist fokal begrenzt, oft in Verbindung mit Nagelveränderungen. Die Pusteln werden im oberen und mittleren Korium von einem perivaskulären und interstitiellen Lymphozyteninfiltrat begleitet.

Charakteristisch für die PPP ist histologisch das Auftreten sogenannter Kogoj'scher Makropusteln, die intraepidermale Ansammlungen neutrophiler Granulozyten darstellen. Gelegentlich sind die ebenfalls neutrophilenreichen sogennanten Munro‐Mikroabszesse, die bei der PV typisch sind, im Stratum corneum zu beobachten (Abbildung 4). Unterhalb der Pustel kann eine Akkumulation von Eosinophilen und Mastzellen beobachtet werden. ^28^, ^58^ Dermal zeigt sich eine gemischtzellig‐lymphozytäre Entzündung, häufig begleitet von erweiterten Kapillargefäßen. Weitere, für die PV charakteristische histologische Merkmale, wie der Verlust des Stratum granulosum, verlängerte Reteleisten/Akanthose sowie Hyperkeratose mit Parakeratose sind nicht immer vorhanden und treten tendenziell eher in älteren und persistierenden Läsionen auf.^2^, ^28^, ^58^, ^68^ In frühen Stadien der PPP findet sich teilweise intraepidermal eine Spongiose und Vesikelbildung, was histologisch der Pompholyx ähneln kann.^2^, ^58^ Bei vesikulären und auch pustulösen Läsionen an Handinnenflächen und Fußsohlen sollte immer eine PAS‐Färbung durchgeführt werden, insbesondere wenn neutrophile Granulozyten in den Vesikeln oder im Stratum corneum vorhanden sind, da Dermatophyteninfektionen die PPP‐ und Pompholyx‐Läsionen imitieren und / oder begleiten können.^69^, ^70^

Histologisch ist die PPP primär durch die Ansammlung neutrophiler Granulozyten innerhalb der Epidermis, die sogenannten Kogoj‐Makropusteln, gekennzeichnet. In frühen Stadien kann das Bild durch intraepidermale Spongiose und Vesikelbildung der Pompholyx (dyshidrosiformes Ekzem) ähneln.

## DIFFERENTIALDIAGNOSEN

Differentialdiagnosen zur PPP schließen die akute PPP, die palmoplantare Psoriasis (ohne Pusteln), die Acrodermatitis continua Hallopeau, das dyshidrosiforme Ekzem mit Pompholyx als Maximalvariante, das chronische Handekzem und die Tinea manus beziehungsweise pedis ein (Abbildung 4).^71^

## THERAPIE DER PPP

Die PPP ist eine chronische Erkrankung, die langfristiger Behandlung und oft Kombinationen topischer sowie systemischer Therapien bedarf. Da es an spezifischen Leitlinien und randomisierten Studien mangelt, orientiert sich die Therapie häufig an der der PV.^72^ Die paradoxe PPP hat im Gegensatz zur PPP eine bessere Prognose. In einer kürzlich erschienenen Metaanalyse von 155 Patienten mit medikamenteninduzierter PPP zeigten 58,8 % der Patienten nach etwa vier Monaten eine komplette Remission, vor allem unter topischen Kortikosteroiden oder nach Wechsel des Biologikums.^73^ Die Wahl einer Systemtherapie bei der paradoxen PPP sollte aufgrund der ebenfalls bestehenden therapiebedürftigen entzündlichen Grunderkrankung interdisziplinär erfolgen.

Topische Therapien der PPP sind hochpotente Kortikosteroide unter Okklusion oder *off label* die Fixkombination von Calcipotriol und Betamethason, wobei auch Januskinase‐Inhibitoren (JAKi) eine *off label* Option darstellen könnten.

### Topische Therapie

Topische Behandlungen sind oft der erste Ansatz bei PPP, stoßen aber aufgrund der geringeren Penetration in das Stratum corneum der Fußsohlen und Handflächen und der Chronizität der PPP an ihre Grenzen. Eine Verbesserung der Penetration hochpotenter topischer Kortikosteroide kann durch Okklusion erreicht werden, dies ist jedoch nur kurzfristig praktikabel.^47^, ^74^, ^75^ Auch wenn Vitamin‐D3‐Analoga wie Maxacalcitol, insbesondere in Kombination mit Betamethason, bei japanischen Patienten mit PPP wirksam war, ist es in Deutschland zur Behandlung der PPP nicht zugelassen.^76^, ^77^ Jedoch kann die fixe Kombination von Betamethason und Calcipotriol, vorzugsweise als Sprühschaum und als auf *polyaphron dispersion* (PAD) Technologie basierter Creme, verwendet werden.^78^, ^79^ Eine weitere Option in der topischen Therapie der PPP könnten die Januskinase‐Inhibitoren (JAKi) sein, bisher ist ein erfolgversprechender Fall einer mit Ruxolitinib therapierten PPP publiziert worden.^80^

Phototherapien sind zeit‐ und ressourcenintensiv. Sie werden oftmals zur Überbrückung bis zum Wirkeintritt einer neu begonnenen Systemtherapie eingesetzt.

### Ultraviolett (UV)‐Strahlung

Die UV‐Strahlung stellt eine unterstützende Behandlungsoption dar. Aufgrund der potenziellen akuten und chronischen Langzeit‐Risiken einer Photochemotherapie ist die Indikation bei chronisch‐rezidivierenden Hauterkrankungen, die wiederholte Bestrahlungszyklen erforderlich machen, etwas strenger zu stellen.^81^

Die Schmalspektrum‐UVB‐Phototherapie zeigte bei japanischen Patienten eine signifikante Verbesserung des PPPASI um 61,4 % nach zwölf Wochen.^82^ Die Standardbehandlung ist jedoch die PUVA‐Photochemotherapie (Psoralen und UVA‐Strahlung), die als Bade‐PUVA für PPP zugelassen ist.^83^ Studien deuten auf einen leichten Vorteil der UVA/PUVA gegenüber UVB‐Strahlung hin.^51^, ^72^, ^81^, ^84^ In der klinischen Praxis kann teilweise ein sehr gutes Therapieansprechen der topischen PUVA beobachtet werden, was allerdings in prospektiven Studien, die auch mit kürzeren Einwirkzeiten (zehn Minuten) arbeiteten, nicht immer bewiesen werden konnte.^85^ Die Kombination von PUVA mit Retinoiden (Re‐PUVA) kann synergistische Effekte erzielen.^86^

Die Excimer‐Lasertherapie (Xenon‐Chlorid‐Laser (XeCl) der Wellenlänge 308 nm) ist eine weitere Therapieoption.^87^ In einer chinesischen prospektiven Vergleichsstudie mit 73 Patienten zeigte der Excimer Laser nach zwölf Wochen mit dreimaliger Anwendung pro Woche ein PPPASI‐75 Ansprechen von 95 % in der Hochdosis‐Gruppe im Vergleich zu 8,3 % und 29,17 % in der Niedrig‐ und Mitteldosis‐Gruppe.^87^, ^88^, ^89^ Es wird von Dosen von mindestens 200 mJ/cm^2^ 1‐ 2‐mal wöchentlich und 10 bis 20 Excimer‐Sitzungen berichtet.^90^, ^91^ Gerade für die TNF‐α induzierte mildere Form der PPP, wo die bestehende Systemtherapie aufgrund anderweitig guter Wirkung fortgeführt werden sollte, könnte dies eine gute Therapieoption darstellen.^33^, ^35^

## SYSTEMTHERAPIE

Von den zur PPP eingesetzten Systemtherapien besitzt nur Acitretin eine Zulassung.^92^ Erfahrungen zu anderen Therapien stammen aus klinischen Studien, aus Beobachtungen des Therapieeffekts, wenn Systemtherapien *in label* für eine Begleitkrankheit verabreicht werden (wie Plaque Psoriasis, Psoriasisarthritis, seronegative Spondylarthropathie oder entzündliche Darmerkrankungen) sowie aus *Off‐label*‐*use*‐Behandlungen.

Acitretin ist die einzig zugelassene Systemtherapie der PPP in Deutschland, sein Einsatz ist aber oft aufgrund von Komorbidität wie zum Beispiel der Hyperlipidämie meist im Rahmen des metabolischen Syndroms, und aufgrund seiner Teratogenität, limitiert. Die anderen konventionellen Systemtherapeutika zeigen ebenfalls Limitationen, obgleich Ciclosporin‐A recht wirkungsvoll sein kann.

### Retinoide

Acitretin ist der einzige Wirkstoff mit expliziter Zulassung für die PPP.^92^ Zwei prospektive Studien mit Acitretin beziehungsweise Etretinat zeigten eine deutliche Reduktion der klinischen Befunde bei Patienten mit PPP nach zwölf Wochen.^51^, ^93^, ^94^ Eine kleine placebokontrollierte Studie mit Alitretinoin, das für das chronische Handekzem zugelassen ist, zeigte bei Patienten mit PPP keine Überlegenheit gegenüber der Placebogruppe.^95^ Wichtige Nebenwirkungen von Retinoiden sind Haut‐/Schleimhauttrockenheit, Hyperlipidämie und möglicherweise Verstärkung einer bestehenden Depression.^92^

Acitretin ist teratogen. Die Anwendung ist bei Frauen, die während der Behandlung oder innerhalb eines Zeitraums von drei Jahren nach Beendigung der Behandlung schwanger werden könnten, kontraindiziert, es sei denn, dass alle Bedingungen des Schwangerschaftsverhütungsprogramms eingehalten werden (siehe entsprechende Fachinformationen).^92^

### Methotrexat (MTX)

Die Datenlage für MTX zur Behandlung der PPP bezüglich prospektiven Studien oder Fallserien ist unzureichend.^97^ Eine retrospektive Analyse mit 42 MTX‐ von insgesamt 201 Behandlungsverläufen zeigte eine durchschnittliche Anwendungsdauer von acht Monaten, die sich in Kombination mit Biologika auf zwölf Monate verlängerte. MTX wird auch in Kombination mit Biologika oder bei begleitender Psoriasisarthritis in Erwägung gezogen.^97^

### Ciclosporin‐A (CsA)

Zwei randomisierte kontrollierte Studien (RCTs) mit CsA bei Patienten mit PPP zeigten eine signifikante Reduktion der Pustelzahl nach einem Monat im Vergleich zu Placebo,^98^, ^99^ (Tabelle 4). Eine einarmige prospektive Studie mit CsA an 48 Patienten in einer Dosierung von 3 mg/kg KG zeigte bei 45 Patienten eine klinische Verbesserung innerhalb von 15–30 Tagen (ein primärer Endpunkt war nicht definiert).^100^ CsA zeichnet sich durch einen schnellen Wirkeintritt aus, jedoch limitieren Nebenwirkungen wie arterielle Hypertonie und Nephrotoxizität die Langzeitbehandlung.^101^ Eine deutsche Analyse zeigte eine exzellente Ansprechrate von 51,4 % für CsA, verglichen mit 19,5 % für Acitretin und 16,8 % für MTX, und ein längeres *Drug Survival* von zwölf Monaten.^97^

**TABELLE 4:** Überblick über prospektive Studien mit *small molecules* und Biologika als Behandlungsmöglichkeiten der palmoplantaren Pustulose (PPP).

Medikament	Anzahl der Patienten in der Verum‐gruppe	Untersuchtes Medikament Klinische Ansprechrate Woche 12–20		Placebo Klinische Ansprechrate Woche 12–20		Literatur
		PPPASI50 (%)	PPPASI75 (%)	PPPASI50 (%)	PPPASI75 (%)	
**Prospektive Placebo‐kontrollierte Studien, die ihren primären Endpunkt erreicht haben**						
Brodalumab 210 mg s.c.	63	54,0	36,0	14,1	8,1	(Okubo et al. 2024)^133^
Guselkumab 100 mg s.c.	54	57,4	20,4	34,0	3,8	(Terui et al. 2019)^126^
Guselkumab 200 mg s.c.	52	36,5	11,5			
Guselkumab 200 mg s.c.	25	60	n. g.	21	n. g.	(Terui et al. 2018)^125^
Risankizumab 150 mg s.c.	61	41	13,1	24,1	15,5	(Okubo et al. 2025)^128^
Apremilast 30 mg bid	46	78,3	43,5	40,9	15,9	(Terui et al. 2023)^104^
Ciclosporin 2,5 mg/kg/Tag*	19	n. g.	n. g.	n. g.	n. g.	(Reitamo 1993)^98^
Ciclosporin 1 mg/kg/Tag*	27	n. g.	n. g.	n. g.	n. g.	(Erkko et al. 1998)^99^
**Prospektive Placebo‐kontrollierte Studien, die ihren primären Endpunkt NICHT erreicht haben**						
Anakinra 100 mg s.c./Tag	31	21 W8	0 W8	16 W8	3 W8	(Cro et al. 2021)^137^
Alitretinoin 30 mg/Tag	24	50	23	67	33	(Reich et al. 2016)^95^
Secukinumab 150 mg s.c.	80	36,5	17,5	34,0	14,1	(Mrowietz et al. 2019 und 2021)^130^, ^131^
Secukinumab 300 mg s.c.	79	52,2	26,6			
Spesolimab s.c. Mehrere Dosierungen	109	n. g.	n. g.	n. g.	n. g.	(Burden et al. 2023)^22^
Ustekinumab 45 mg s.c.	15	13,3	n. g.	37,5	n. g.	(Bissonnette et al. 2014)^63^
**Prospektive Placebo‐kontrollierte Studien mit signifikantem Effekt aber ohne eindeutig definierten primären Endpunkt**						
Etanercept 50 mg s.c.	10	n. g.	n. g.	n. g.	n. g.	(Bissonnette et al. 2008)^119^

Prospektive einarmige klinische Studien bei der palmoplantaren Pustulose, die den primären Endpunkt erreicht haben*				
**Medikament**	**Anzahl der Patienten**	**PPPASI50 (%)**	**PPPASI75 (%)**	**Literatur**
Guselkumab 100 mg s.c.	50	66 (W24)	34 (W24)	(Wilsmann‐Theis et al. 2025)^127^
Apremilast 30 mg bid	20	61,9 (W20)	14,3 (W20)	(Wilsmann‐Theis et al. 2021)^103^
bid: zweimal täglich; PPPASI: Palmoplantar Pustulosis Area and Severity Index; s.c. subkutan; W: Woche *Nur Studien mit mehr als 12 Patienten wurden berücksichtigt				

*Abk*.: s.c. = subkutan; n.g. = nicht genannt; bid = bis in die (zweimal täglich); W = Woche; *primärer Endpunkt wurde erreicht; +nicht placebokontrolliert

### Fumarsäureester

In einer klinischen Studie mit Fumarsäureestern mit 13 Patienten erreichten acht Patienten an Woche 24 eine Reduktion des klinischen Scores für die PPP um 49 % an den Händen und um 44 % an den Füßen.^102^ Als häufige limitierende Nebenwirkung gelten gastrointestinale Beschwerden und Flush, auch der verlangsamte Wirkeintritt schränkt Fumarsäureester zur Behandlung ein.

Moderne *small molecules* wie Apremilast und Januskinase‐Inhibitoren könnten sich bei der PPP als wirkungsvoller erweisen als bei der PV.

### Neue *small molecules*

#### Apremilast, Phosphodiesterase (PDE)‐4 Inhibitor

Apremilast wurde in Studien in der Standarddosierung (mit Standard‐Aufdosierung) bei Patienten mit PPP eingesetzt: Eine multizentrische einarmige Studie in Deutschland an 20 Patienten konnte bei 61,9 % der Patienten nach 20 Therapiewochen eine PPPASI‐50 Reduktion erreichen sowie eine mediane Reduktion des PPPASI um 57,1 %.^103^ Eine japanische placebokontrollierte Studie mit 90 Teilnehmern bestätigte diese guten Ergebnisse und zeigte ebenfalls einen schnellen Rückgang der Pustelzahl (Tabelle 4). An Woche 16 lag die mittlere PPPASI Reduktion in der Apremilastgruppe bei 64,3 % vs. 42,4 % im Placebo‐Arm. Es wurden ferner knapp 40 Patienten in Fallserien und Fällen mit überwiegend positiven Therapieansprechen beschrieben.^105^, ^106^, ^107^, ^108^, ^109^

### Januskinase (JAK)‐Inhibitoren

Zu JAK‐Inhibitoren gibt es mehrere vielversprechende Fallberichte und Fallserien vor allem mit Upadacitinib, Tofacitinib und Baricitinib.^110^, ^111^, ^112^, ^113^, ^114^, ^115^, ^116^ In einer deutschen Fallserie mit fünf überwiegend therapierefraktären Patienten kam es zu einem schnellen Ansprechen innerhalb der ersten vier Wochen.^117^ Allerdings gebieten Empfehlungen der EMA bei Patienten ab 65 Jahren, bei Personen mit einem erhöhten Risiko für schwerwiegende kardiovaskuläre Ereignisse (wie Herzinfarkt oder Schlaganfall), bei Personen, die rauchen oder in der Vergangenheit über einen längeren Zeitraum geraucht haben sowie bei Personen mit einem erhöhten Krebsrisiko, JAK‐Inhibitoren nur dann therapeutisch einzusetzen, wenn es keine geeigneten Behandlungsalternativen gibt. JAK‐Inhibitoren sollten bei Patienten mit Risikofaktoren für Blutgerinnsel in der Lunge und in tiefen Venen (venöse Thromboembolien (VTE)), die über die oben genannten hinausgehen, mit Vorsicht angewendet werden. Darüber hinaus sollten die Dosen nach Möglichkeit bei Patientengruppen reduziert werden, die ein erhöhtes Risiko für VTE, Krebs oder schwerwiegende kardiovaskuläre Ereignisse aufweisen.^118^

### Biologika‐Therapie

In den letzten Jahren wurden durch den Einsatz von TNF‐, IL‐23‐ und IL‐17‐Inhibitoren bedeutende Fortschritte bei der Behandlung der PPP erzielt, wobei die Erfolgsraten jedoch nicht so hoch sind wie bei der PV.

Unter den Biologika zeigen insbesondere Interleukin‐23 und Interleukin‐17RA‐Blocker in klinischen Studien eine gute Wirksamkeit in der Behandlung der PPP. Andere Zytokin‐Inhibitoren wie Ustekinumab, Spesolimab oder IL‐1‐Inhibitoren erzielten hingegen uneinheitliche oder überwiegend unzureichende Wirksamkeitsnachweise.

### TNF‐Blocker

Die Datenlage zu TNF‐Blockern bei PPP ist begrenzt, möglicherweise aufgrund der Gefahr paradoxer Reaktionen mit pustulösen palmoplantaren Veränderungen bei deren Anwendung in anderen Indikationen.^32^ Eine kleine kanadische placebokontrollierte Studie mit Etanercept zeigte eine signifikante PPPASI Reduktion nach 24 Wochen in der reinen Etanercept‐Gruppe im Vergleich zur Baseline. Nach zwölf Wochen Therapie mit Etanercept – hier auch im Vergleich zum Placebo – war kein Nutzen zu erkennen (Tabelle 4).^119^

### Ustekinumab (IL12/23‐Blockade)

Ustekinumab wird häufig bei paradoxen Reaktionen auf TNF‐Blocker eingesetzt.^120^ Die Studienlage zur PPP ist uneinheitlich. Eine placebokontrollierte Studie zeigte keinen Vorteil, während eine offene Studie in den USA bei einer Mehrheit der Patienten eine Verbesserung feststellte. Fallberichte unterstreichen die variierenden Beobachtungen, wobei Therapiedauer und Dosis möglicherweise eine Rolle spielen.^38^, ^63^, ^121^

### IL23p19‐Blocker

Unter den IL‐23‐Blockern gibt es die beste Datenlage zu Guselkumab (Tabelle 4), das 2018 in Japan zur Therapie der PPP zugelassen wurde,^122^, ^123^ gefolgt von Risankizumab.^124^

Für Guselkumab belegen drei randomisierte, placebokontrollierte Studien in Japan eine signifikante und anhaltende Verbesserung der PPPASI‐Werte sowie der Lebensqualität bis Woche 52. Eine deutsche multizentrische, offene Studie mit 50 Patienten mit PPP bestätigte diese Ergebnisse, ohne Unterschiede im Ansprechen bezüglich Krankheitsdauer, Raucherstatus, Körpergewicht, Geschlecht oder begleitender Psoriasis festzustellen (Tabelle 4).^125^, ^126^, ^127^

Kürzlich erschienen ist eine japanische Studie zu Risankizumab bei PPP. Es zeigten 41 % der mit Risankizumab therapierten Patienten (n = 61) ein PPPASI‐50 Ansprechen von 41 % versus 24.1 % der Placebo‐behandelten Patienten (n = 58) an Woche 16. Ein signifkanter Effekt wurde bei der PPPASI‐75 Antwort nicht erreicht.^128^ Eine retrospektive Analyse von 16 Patienten, die mit IL‐23‐Blockern (Guselkumab, Tildrakizumab, Risankizumab) behandelt wurden, zeigte ebenfalls positive Ergebnisse mit PPPASI‐50‐ und PPPASI‐75‐Reduktionen.^129^

### IL‐17‐ UND IL‐17RA‐BLOCKER

#### Secukinumab (IL‐17A‐Antagonist)

Die 52‐wöchige RCT 2PRECISE‐Studie zu Secukinumab zeigte nach 16 Wochen keine Überlegenheit gegenüber Placebo. Eine deutliche Verbesserung war nach 52 Wochen unter Secukinumab Therapie zu sehen. In der Verlängerungsstudie über 148 Wochen wurde eine weitere Verbesserung der PPP erzielt.^130^, ^131^ Eine spanische retrospektive Kohortenstudie zeigte ein PPPASI‐75‐Ansprechen bei über 50 % der Patienten nach zwei Jahren Behandlung (Tabelle 4). ^132^

#### Brodalumab (IL‐17‐Rezeptor A (IL17RA) Antagonist)

Eine placebokontrollierte Phase‐3‐Studie (RCT) mit 126 Patienten mit PPP zeigte nach 16 Wochen eine signifikante Überlegenheit von Brodalumab gegenüber Placebo bezüglich PPPASI‐50‐ und PPPASI‐75‐Ansprechen. Die Ergebnisse der offenen 52‐wöchigen Phase wurden noch nicht publiziert,^133^ (Tabelle 4).

#### Weitere IL‐17 Blocker

Bimekizumab, das gezielt die Untereinheiten IL‐17 A und F blockiert, führte in einer retrospektiven Analyse bei 21 Patienten mit PPP in Frankreich bei 17 Patienten nach 1–4 Monaten Therapie zur Erscheinungsfreiheit.^134^ Eine prospektive placebokontrollierte Studie mit Bimekizumab (NCT07219420) bei PPP startet in nächster Zukunft (https://clinicaltrials.gov/search?cond=palmoplantar%20pustulosis&intr=Bimekizumab). Eine chinesische Studie mit dem IL‐17A‐Antagonisten Ixekizumab zeigte schnelles Ansprechen bei intraläsionaler Gabe, die Methode gilt jedoch als nicht praxistauglich.^135^

### Spesolimab (IL36 Rezeptorantagonist)

Spesolimab, ein humaner monoklonaler Antikörper gegen den IL‐36R, erreichte in einer europäischen placebokontrollierten Phase IIa Studie nicht den primären Endpunkt, der mit Erreichen des PPPASI50 an Woche 16 definiert war,^136^ (Tabelle 4).

### IL‐1‐Inhibitoren

Anakinra, ein IL‐1‐Rezeptorantagonist, zeigte in einer placebokontrollierten Studie bei PPP keine Überlegenheit gegenüber Placebo, und in zwei Fallberichten wurde die Behandlung aufgrund fehlender Wirkung abgebrochen,^137^, ^138^ (Tabelle 4).

### Dupilumab

Dupilumab, ein monoklonaler Antikörper gegen die Alpha‐Untereinheit der Rezeptoren von Interleukin‐4 und Interleukin‐13, ist zur Behandlung der mittelschweren bis schweren atopischen Dermatitis und der Prurigo nodularis zugelassen. In einer prospektiven Studie mit Dupilumab aus China bei Patienten mit PPP ohne Psoriasis in der Eigen‐ oder Familienanamnese erreichten neun der zehn Patienten nach 16 Wochen ein PPPASI‐75‐Ansprechen.^139^

Vielversprechende neue Therapieansätze ergeben sich aus einer Fallserie zu Dupilumab.^139^ Zudem könnten zukünftige Studien zu JAK‐ und TYK2‐Inhibitoren sowie zu Bimekizumab weitere Fortschritte bringen, da hierzu bereits einzelne erfolgreiche Behandlungsfälle und Fallserien publiziert wurden.

### Weitere Studien und Register

Weitere Studien zur Behandlung der PPP sind notwendig. Zum aktuellen Zeitpunkt sind gemäß den Angaben von *ClincalTrials.gov* eine prospektive Studie zu Deucravacitinib, einem Tyk2‐Inhibitor derzeit in den USA (NCT05710185) rekrutierend und in China (NCT07000630) geplant.^140^

Erste Studien zu topischen JAK‐Inhibitoren wie dem pan‐JAK Inhibitor Delgocitinib sind in Vorbereitung (NCT07013201). Der IL‐36‐Rezeptor‐Antagonist Imsidolimab ist in der klinischen Entwicklung (NCT03633396).^141^ Zum Sonelokimab, einem bispezifschen Nanobody, der IL‐17A und IL‐17F inhibitiert, wurde eine placebokontrollierte Phase II Studie zur PPP in Deutschland durchgeführt (https://euclinicaltrials.eu/ctis‐public/view/2024‐513305‐32‐00?lang=en). Ein japanisches Register https://clinicaltrials.gov/study/NCT04459507 soll zukünftig weitere Informationen zu dieser komplexen Erkrankung liefern, ähnlich wie das deutsche Register ppBest (https://www.ppbest.de).

Neben der medikamentösen Therapie ist für den Behandlungserfolg die Optimierung des Lebensstils unabdingbar.

## MANAGEMENT DER PPP

Beim Management der PPP sollten Provokationsfaktoren erkannt und gemieden beziehungsweise behandelt werden,^142^ (siehe Checkliste Tabelle 5). So sollten Betroffene über die Bedeutungvon Nichtrauchen und Nikotinkarenz informiert werden. Die positiven Effekte von Nichtrauchen auf den Verlauf der PPP wurden in Studien bestätigt.^143^ Auch Übergewicht zählt zu den Risikofaktoren der PPP, daher sollte, vor allem bei Nichtansprechen auf die empfohlenen Therapien, ein aktives Gewichtsmanagement erwogen werden. Bei der Wahl der Systemtherapie müssen Begleiterkrankungen berücksichtigt werden, und das Management sollte dann interdisziplinär erfolgen.

**TABELLE 5:** Checkliste: Management von Risiko‐ und/oder Provokationsfaktoren der palmoplantaren Pustulose. Die Checkliste bietet eine strukturierte Übersicht über die wichtigsten Empfehlungen zum Management der palmoplantaren Pustulose. In drei zentralen Abschnitten werden potenzielle Provokationsfaktoren, berufliche Aggravation sowie relevante Komorbiditäten systematisch erfasst und praxisnahe Maßnahmen aufgeführt.

Empfehlungen		Maßnahmen (Beispiele)
**1. Eruierung möglicher Provokationsfaktoren** ∙ Tabakrauchen	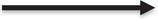	Nikotinkarenz (Raucherentwöhnungsprogramm)
∙ Übergewicht	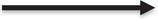	Gewichtsreduktion (Ernährungsberatung, sportliche Aktivität)
∙ Infektiöse Foci	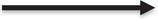	Sanierung nach fachärztlicher Maßgabe (z. B. HNO‐ und Zahnbereich)
∙ Mechanische Reize	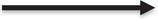	Meiden soweit möglich
∙ Psychologischer Stress	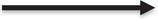	Stressreduktion (psychologische Unterstützung)
∙ Medikamente: Paradoxe PPP (z. B. durch TNF‐ Inhibitoren)	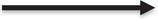	Umsetzen erwägen
**2. Prüfung einer beruflichen Aggravation**	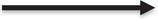	Meldung an die Berufsgenossenschaft (Einleitung Hautarztverfahren)
**3. Screening auf Komorbidität** Knochen‐ und Gelenkbeteiligung Psoriasis vulgaris Metabolisches Syndrom (Diabetes, arterielle Hypertonie, Hyperlipidämie, Übergewicht) Psychiatrische Erkrankungen		

## ZUSAMMENFASSUNG ZUR THERAPIE DER PPP

**ABBILDUNG 5:**
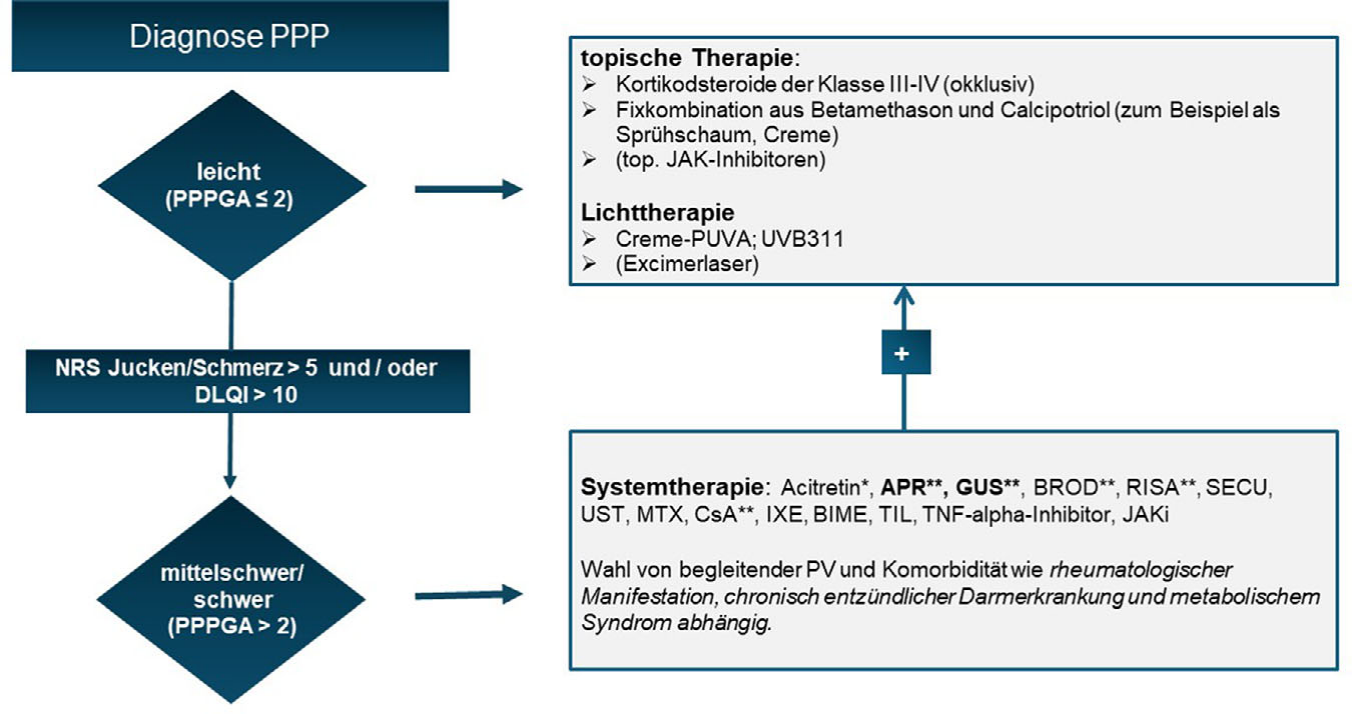
Therapiealgorithmus der palmoplantaren Pustulose (PPP). Der Algorithmus zeigt ein vereinfachtes Schema zum therapeutischen Vorgehen bei der PPP abhängig vom Schweregrad und eventuellen Begleiterkrankungen. Liegt eine begleitende Knochen/Gelenkbeteiligung oder Psoriasis vulgaris vor, erfolgt die Systemtherapie entsprechend dem Gelenk‐ und Hautbefall sowie bestehender Komorbidität. Die Behandlung richtet sich zudem nach dem Schweregrad der PPP. Bei mittelschwerer bis schwerer Ausprägung als auch bei leichter Ausprägung mit Vorliegen eines DLQI > 10 und/oder NRS > 5 ist eine systemische Therapie angezeigt. *Zugelassene Therapie für PPP auch ohne Vorliegen von PV; **RCT bei PPP mit Erreichen des primären Endpunktes vorhanden; fettgedruckte Systemtherapie = vorzugsweise einzusetzen, da höchste Evidenz. *Abkürzungen*: Psoralen + UVA‐Phototherapie; UVB311 = UVB‐Phototherapie mit 311 nm; PV = Psoriasis vulgaris/Plaque‐Psoriasis; ADA = Adalimumab; APR = Apremilast; BIME = Bimekizumab; BROD = Brodalumab; CsA = Ciclosporin A; GUS = Guselkumab; IXE = Ixekizumab; MTX = Methotrexat; RISA = Risankizumab; SECU = Secukinumab; TIL = Tildrakizumab; TNF = Tumor‐Nekrose Faktor; UST = Ustekinumab.

Die Therapie der PPP stellt immer noch eine große Herausforderung dar. Topische hochpotente Steroide unter Okklusion gehören zur Standardtherapie, allein oder begleitend zu einer Phototherapie und/oder Systemtherapie (Abbildung 5). Insbesondere die topische PUVA‐Photochemotherapie nimmt im Therapiefeld der PPP einen festen Platz ein. UV‐Phototherapien sind für die Betroffenen zeitintensiv und nicht für die Langzeitbehandlung geeignet. Zur Systemtherapie der PPP ist nur Acitretin zugelassen, welches aufgrund des langsamen Wirkeintritts und des ungünstigen Nebenwirkungsprofils bei Patienten mit metabolischem Syndrom sowie der obligaten Teratogenität oft nicht zum Einsatz kommen kann oder abgesetzt werden muss. Ciclosporin zeigt hier einen schnelleren und effektiveren Wirkeintritt, ist aber keine gute Option zur Langzeittherapie. MTX hat in den beschriebenen Analysen zur PPP nicht überzeugt. Apremilast überzeugte hingegen in einer japanischen und einer deutschen prospektiven Studie. Gerade bei der Pustelreduktion beweist es eine besondere Wirkstärke. Weitere s*mall molecules* wie die JAK‐Inhibitoren zeigten sich in ersten Fallberichten und Fallserien wirksam. Biologika wie IL‐23‐ oder IL‐17RA‐Inhibitoren zeigen teilweise gute Ansprechraten (Tabelle 4). Eine Zulassung in Japan besitzt derweil der IL‐23‐Blocker Guselkumab. In Hinblick auf die Patientenführung ist es unter Guselkumab wichtig zu vermerken, dass die Daten zur PPPASI Besserung in den frühen Messzeiten von den 1 oder 2 Jahresdaten übertroffen werden. Der IL‐36‐ Rezeptor Antagonist Spesolimab oder der IL1‐Inhibitor Anakinra konnten die Hoffnungen bei der PPP nicht erfüllen. Weitere Studien werden benötigt, um das Wirkprofil von Biologika mit anderen Angriffspunkten wie zum Beispiel IL17 A/F zu evaluieren.

## DANKSAGUNG

Open access Veröffentlichung ermöglicht und organisiert durch Projekt DEAL.

## INTERESSENKONFLIKT

Die folgenden Autoren waren beratend tätig und/oder erhielten Honorare oder Reisekostenerstattungen für Referenten und/oder erhielten Zuschüsse und/oder nahmen an klinischen Studien der folgenden Unternehmen teil:

Tanja Fetter: Biogen, AstraZeneca, Incyte, ADF

Dagmar Wilsmann‐Theis war Beraterin, Referentin oder Prüfärztin für Studien von Abbvie, Almirall, Amgen, Biogen, Boehringer Ingelheim, Bristol Myers Squibb, Celgene, GlaxoSmithKline, Hexal, Incyte, Janssen‐Cilag, Leo Pharma, Eli Lilly, Medac, Merck Sharp & Dohme Corp., Moonlake, Novartis, Pfizer and UCB Pharma.

Ulrich Mrowietz war als honorierter Berater und/oder Redner für folgende Firmen tätig: AbbVie, Aditxt, Almirall, Amgen, Biogen, Boehringer‐Ingelheim, Bristol‐Myers Squibb, Eli Lilly, Immunic, Janssen‐Cilag, LEO Pharma, Merck, Sharp & Dohme, Novartis, Phi‐Stone, SelectION, UCB Pharma, UNION therapeutics.

Rotraut Mössner war als honorierte Beraterin und/oder Rednerin und/oder Empfängerin von Forschungsunterstüzungen und/oder Teilnehmerin an klinischen Studien für folgende Firmen tätig: AbbVie, Amgen, Almirall, Biogen IDEC, Böhringer‐Ingelheim, Celgene, Janssen‐Cilag, Leo Pharma, Lilly, Moonlake, MSD SHARP & DOHME, Novartis Pharma, Pfizer and UCB.

Robert Sabat erhielt Forschungszuschüsse oder Verträge für klinische Studien von AbbVie, Boehringer Ingelheim Pharma, Celgene/Amgen, Celgene/Bristol Myers Squibb, Charité Research Organization, CSL Behring, ICON, IQVIA RDS, Incyte, Janssen‐Cilag/Janssen Research & Development, MoonLake Immunotherapeutics, Novartis, Parexel, Rheinischen Friedrich‐Wilhelms‐Universität Bonn, Sanofi Aventis, TFS und UCB Biopharma, die seiner Einrichtung gezahlt wurden; Honorare für Vorträge, Beratung oder die Teilnahme an Beiräten von AbbVie, Almirall Hermal, Amgen, Bayer Schering Pharma, Bruno Bloch Stiftung, Janssen‐Cilag/ Janssen Research & Development, Novartis, UCB Biopharma, Universitätsmedizin Greifswald und Wundnetz Berlin‐Brandenburg; außerdem ist er Mitglied des International Psoriasis Council (unbezahlt) und Sprecher der Psoriasis‐Arbeitsgruppe der Arbeitsgemeinschaft Dermatologische Forschung (ADF) (unbezahlt).

Alle anderen Autoren erklären, dass die Forschung in Abwesenheit jeglicher kommerziellen oder finanziellen Interessen und Beziehungen durchgeführt wurde, die als potenzieller Interessenkonflikt ausgelegt werden könnten.

## FINANZIERUNG

Tanja Fetter erhielt eine Förderung durch das BONFOR‐Programm der Universität Bonn (Gerok‐Stelle, Förderkennzeichen 2023‐1A‐17). Die anderen Autoren erklären, dass sie keine finanzielle Unterstützung für die Forschung, Autorenschaft und/oder Veröffentlichung dieses Artikels erhalten haben.

## [CME Questions/Lernerfolgskontrolle]

1. Welche Kurzbeschreibung trifft auf die palmoplantare Pustulose (PPP) zu?
  - Sie ist eine akute, selbstlimitierende Erkrankung
  - Sie ist genetisch identisch mit der Psoriasis vulgaris
  - Sie manifestiert sich mit sterilen Pusteln an Handflächen und Fußsohlen
  - Männer und Frauen sind etwa gleichhäufig betroffen
  - Es gibt aktuelle Leitlinien für die Therapie
2. Welche der folgenden Erkrankungen sollte bei der Diagnose einer palmoplantaren Pustulose (PPP) differenzialdiagnostisch in erster Linie in Betracht gezogen und ggf. ausgeschlossen werden?
  - Vitiligo
  - Tinea manus/pedis
  - Lupus erythematodes
  - Rosacea
  - Erythema exsudativum multiforme
3. Welcher der folgenden Faktoren gilt als der wichtigste Provokationsfaktor für die palmoplantare Pustulose (PPP)?
  - Warme Bäder
  - Mangel an Vitamin D
  - Regelmäßiger Konsum von Alkohol
  - Tabakrauchen
  - UV‐Exposition
4. Eine „paradoxe PPP” tritt am häufigsten in Zusammenhang mit der Behandlung welcher Medikamentenklasse auf?
  - Retinoide
  - Ciclosporin
  - TNF‐α Blocker
  - Fumarsäureester
  - Topische Kortikosteroide
5. Welche Aussage zu Knochen/Gelenkbeteiligung bei der PPP trifft zu?
  - Eine PPP ist ein notwendiges Kriterium für das Vorliegen einer pustulösen Arthroosteitis
  - Eine PPP ist ein notwendiges Kriterium für das Vorliegen eines SAPHO‐Syndroms
  - Patienten mit PPP mit einer nichtinfektiösen Arthritis mehrerer Fingergelenke erfüllen immer die CASPAR‐Kriterien für eine Psoriasisarthritis
  - Bei Patienten mit PPP liegt häufig ein positiver Rheumafaktor vor
  - Eine sternokostoklavikulärer Hyperostose ist ***keine*** typische Manifestation der Knochen/Gelenkbeteiligung bei der PPP
6. Welche Aussage trifft auf die genetische Basis der PPP zu?
  - Sie ist eng mit dem PSORS1‐Locus assoziiert
  - Sie unterscheidet sich genetisch deutlich von der Psoriasis vulgaris, insbesondere fehlt die PSORS1‐Assoziation
  - Sie ist genetisch überwiegend durch Mutationen im *IL36RN*‐Gen bedingt
  - Sie ist durch eine autosomal‐dominante Vererbung gekennzeichnet
  - Die Genetik der PPP ist vollständig aufgeklärt und zeigt keine Unterschiede zur GPP
7. Welche Immunmediatoren spielen eine wichtige Rolle in der Pathogenese der palmoplantaren Pustulose (PPP), die durch aktivierte Keratinozyten sezerniert werden und neutrophile Granulozyten anlocken?
  - Interleukin‐4 (IL‐4) und IL‐13
  - Chemokine CXCL6 und CXCL8
  - Interferon‐gamma (IFN‐γ) und IFN‐α
  - Interleukin‐10 (IL‐10) und IL‐31
  - Chemokine CXCL9 und CXCL11
8. Welche Aussage trifft am besten auf die Pathogenese der PPP zu?
  - Sie beginnt im Bereich der Haarfollikel mit T‐Zell‐Aktivierung
  - Sie beginnt um das Acrosyringium, dem intraepidermalen Ausgangskanal der Schweißdrüsen, mit neutrophiler Infiltration
  - Sie ist eine direkte Folge einer Pilzinfektion der Haut
  - Sie entsteht durch eine autoimmune Reaktion gegen Keratinozyten
  - Die Pautrier‐ Mikroabszesse sind typisch für die PPP
9. Welche Aussage beschreibt die Wirksamkeit der UV‐Therapie bei PPP am besten?
  - UV‐B ist effektiver als PUVA bei der Behandlung der PPP
  - Die Bade PUVA‐Therapie ist zur Therapie der PPP zugelassen
  - UV‐Therapie ist bei PPP kontraindiziert
  - UV‐Therapie ist nur bei Psoriasis vulgaris wirksam, nicht bei PPP
  - Excimer‐Therapie darf bei der PPP nicht eingesetzt werden
10. Welche systemische Therapie ist in Deutschland für die Behandlung der PPP zugelassen?
  - Methotrexat
  - Ciclosporin
  - Acitretin
  - Apremilast
  - Guselkumab

Liebe Leserinnen und Leser, der Einsendeschluss an die DDA für diese Ausgabe ist der 30. Juni 2026.

Die richtige Lösung zum Thema Einsatz von nichtinvasiver optischer Bildgebung in der Dermatologie in Heft 11/2025 ist: 1b, 2c, 3a, 4a, 5c, 6b, 7c, 8c, 9c, 10b

Bitte verwenden Sie für Ihre Einsendung das aktuelle Formblatt auf der folgenden Seite oder aber geben Sie Ihre Lösung online unter http://jddg.akademie-dda.de ein.

## References

[1] Andrews GC , Birkman FW , Kelly RJ . Recalcitrant pustular eruptions of the palms and soles. (Arch. of Derm. and Syph., April, 1934, xxix, p. 549.). Br J Dermatol. 1935;47(11):487.

[2] Masuda‐Kuroki K , Murakami M , Kishibe M , et al. Diagnostic histopathological features distinguishing palmoplantar pustulosis from pompholyx. J Dermatol. 2019;46(5):399‐408. 30919463 10.1111/1346-8138.14850

[3] Sachs W , Scannone F . So‐called pustular psoriasis. J Invest Dermatol. 1945;6:349‐354. 21010972 10.1038/jid.1945.33

[4] Twelves S , Mostafa A , Dand N , et al. Clinical and genetic differences between pustular psoriasis subtypes. J Allergy Clin Immunol. 2019;143(3):1021‐1026. 30036598 10.1016/j.jaci.2018.06.038 PMC6403101

[5] Weisenseel P , Wilsmann‐Theis D , Kahl C et al. Pustulöse Psoriasis. Hautarzt. 2016;67(6):445‐453. 27240667 10.1007/s00105-016-3804-4

[6] Navarini AA , Burden AD , Capon F , et al. European consensus statement on phenotypes of pustular psoriasis. J Eur Acad Dermatol Venereol. 2017;31(11):1792‐1799. 28585342 10.1111/jdv.14386

[7] Yamamoto T . Similarity and difference between palmoplantar pustulosis and pustular psoriasis. J Dermatol. 2021;48(6):750‐760. 33650702 10.1111/1346-8138.15826

[8] Brunasso AMG , Massone C . Clinical characteristics, genetics, comorbidities and treatment of palmoplantar pustulosis: A different perspective. J Dermatol. 2021;48(1):e47. 33211364 10.1111/1346-8138.15644

[9] Brunasso AMG , Puntoni M , Aberer W , Delfino C et al. Clinical and epidemiological comparison of patients affected by palmoplantar plaque psoriasis and palmoplantar pustulosis: a case series study. Br J Dermatol. 2013;168(6):1243‐1251. 23301847 10.1111/bjd.12223

[10] Murakami M , Ohtake T , Horibe Y , et al. Acrosyringium is the main site of the vesicle/pustule formation in palmoplantar pustulosis. J Invest Dermatol. 2010;130(8):2010‐2016. 20393482 10.1038/jid.2010.87

[11] Hiraiwa T , Yamamoto T . Nail involvement associated with palmoplantar pustulosis. Int J Dermatol. 2017;56(2):e28‐e29. 27808399 10.1111/ijd.13224

[12] Murakami M , Terui T . Palmoplantar pustulosis: Current understanding of disease definition and pathomechanism. J Dermatol Sci. 2020;98(1):13‐19. 32201085 10.1016/j.jdermsci.2020.03.003

[13] Kharawala S , Golembesky AK , Bohn RL , Esser D . The clinical, humanistic, and economic burden of palmoplantar pustulosis: a structured review. Expert Rev Clin Immunol. 2020;16(3):253‐266. 32073337 10.1080/1744666X.2019.1708194

[14] Trattner H , Blüml S , Steiner I , et al. Quality of life and comorbidities in palmoplantar pustulosis ‐ a cross‐sectional study on 102 patients. J Eur Acad Dermatol Venereol. 2017;31(10):1681‐1685. 28252813 10.1111/jdv.14187

[15] Burge SM , Ryan TJ . Acute palmoplantar pustulosis. Br J Dermatol. 1985;113(1):77‐83. 4015972 10.1111/j.1365-2133.1985.tb02046.x

[16] Cheng A , Deng X , Yang F , et al. Treatment Patterns and Negative Health Outcomes in Palmoplantar Pustulosis Patients in Germany and the US. Dermatol Ther (Heidelb). 2024;14(3):627‐641. 38441820 10.1007/s13555-024-01109-z PMC10965862

[17] Kubota K , Kamijima Y , Sato T , et al. Epidemiology of psoriasis and palmoplantar pustulosis: a nationwide study using the Japanese national claims database. BMJ Open. 2015;5(1):e006450. 10.1136/bmjopen-2014-006450 PMC4298108 25588781

[18] Heidemeyer K , Cazzaniga S , Dondi L , et al. Variables associated with joint involvement and development of a prediction rule for arthritis in patients with psoriasis. An analysis of the Italian PsoReal database. J Am Acad Dermatol. 2023;89(1):53‐61. 36965671 10.1016/j.jaad.2023.02.059

[19] Andersen YMF , Augustin M , Petersen J , et al. Characteristics and prevalence of plaque psoriasis in patients with palmoplantar pustulosis. Br J Dermatol. 2019;181(5):976‐982. 30815849 10.1111/bjd.17832

[20] Wilsmann‐Theis D , Jacobi A , Frambach Y , et al. Palmoplantar pustulosis – a cross‐sectional analysis in Germany. Dermatol Online J. 2017;23(4):1‐11, 13030/qt0h15613d. 28541870

[21] Bhushan M , Burden AD , MCelhone K , et al. Oral liarozole in the treatment of palmoplantar pustular psoriasis: a randomized, double‐blind, placebo‐controlled study. Br J Dermatol. 2001;145(4):546‐553. 11703279 10.1046/j.1365-2133.2001.04411.x

[22] Burden AD , Bissonnette R , Navarini AA , et al. Spesolimab Efficacy and Safety in Patients with Moderate‐to‐Severe Palmoplantar Pustulosis: A Multicentre, Double‐Blind, Randomised, Placebo‐Controlled, Phase IIb, Dose‐Finding Study. Dermatol Ther (Heidelb). 2023;13(10):2279‐2297. 37731086 10.1007/s13555-023-01002-1 PMC10539230

[23] Benzian‐Olsson N , Dand N , Chaloner C , et al. Association of Clinical and Demographic Factors With the Severity of Palmoplantar Pustulosis. JAMA Dermatol. 2020;156(11):1216‐1222. 32936291 10.1001/jamadermatol.2020.3275 PMC7495329

[24] Michaëlsson G , Gustafsson K , Hagforsen E . The psoriasis variant palmoplantar pustulosis can be improved after cessation of smoking. J Am Acad Dermatol. 2006;54(4):737‐738. 16546609 10.1016/j.jaad.2005.07.024

[25] Takahara M , Hirata Y , Nagato T , et al. Treatment outcome and prognostic factors of tonsillectomy for palmoplantar pustulosis and pustulotic arthro‐osteitis: A retrospective subjective and objective quantitative analysis of 138 patients. J Dermatol. 2018;45(7):812‐823. 29732605 10.1111/1346-8138.14348

[26] Kouno M , Nishiyama A , Minabe M , et al. Retrospective analysis of the clinical response of palmoplantar pustulosis after dental infection control and dental metal removal. J Dermatol. 2017;44(6):695‐698. 28150339 10.1111/1346-8138.13751

[27] Yamamoto T , Yokozeki H , Tsuboi R . Koebner's phenomenon associated with palmoplantar pustulosis. J Eur Acad Dermatol Venereol. 2007;21(7):990‐992. 17659020 10.1111/j.1468-3083.2006.02065.x

[28] Eriksson H , Lundin M . Palmoplantar pustulosis: a clinical and immunohistological study. Br J Dermatol. 1998;138(3):390‐398. 9580788 10.1046/j.1365-2133.1998.02113.x

[29] Obermeyer L , Skudlik C , John SM , Brans R . Berufsdermatologische Aspekte der Pustulosis palmoplantaris : Diskussionsbeitrag anhand einer retrospektiven Datenauswertung. Hautarzt. 2020;71(9):699‐704. 32430542 10.1007/s00105-020-04611-5

[30] Brunasso Vernetti AMG , Puntoni M , Massone C . Palmoplantar Pustulosis and Allergies: A Systematic Review. Dermatol Pract Concept. 2019;9(2):105‐110. 31106012 10.5826/dpc.0902a05 PMC6502300

[31] Masui Y , Ito A , Akiba Y , et al. Dental metal allergy is not the main cause of palmoplantar pustulosis. J Eur Acad Dermatol Venereol. 2019;33(4):e180‐e181. 30653749 10.1111/jdv.15434

[32] Shmidt E , Wetter DA , Ferguson SB , Pittelkow MR . Psoriasis and palmoplantar pustulosis associated with tumor necrosis factor‐α inhibitors: the Mayo Clinic experience, 1998 to 2010. J Am Acad Dermatol. 2012;67(5):e179‐85. 21752492 10.1016/j.jaad.2011.05.038

[33] Maronese CA , Valenti M , Moltrasio C , et al. Paradoxical Psoriasis: An Updated Review of Clinical Features, Pathogenesis, and Treatment Options. J Invest Dermatol. 2024;144(11):2364‐2376. 38958610 10.1016/j.jid.2024.05.015

[34] Mössner R , Pinter A . Paradoxical palmoplantar pustulosis induced by secukinumab and brodalumab: a report of three cases. Eur J Dermatol. 2020. 10.1684/ejd.2020.3702 32293563

[35] Navarro R , Daudén E . Clinical management of paradoxical psoriasiform reactions during TNF‐ α therapy. Actas Dermosifiliogr. 2014;105(8):752‐761. 23938073 10.1016/j.ad.2013.05.007

[36] Xie W , Xiao S , Huang H , Zhang Z . Incidence of and Risk Factors for Paradoxical Psoriasis or Psoriasiform Lesions in Inflammatory Bowel Disease Patients Receiving Anti‐TNF Therapy: Systematic Review With Meta‐Analysis. Front Immunol. 2022;13:847160. 35300336 10.3389/fimmu.2022.847160 PMC8921985

[37] Conrad C , Di Domizio J , Mylonas A , et al. TNF blockade induces a dysregulated type I interferon response without autoimmunity in paradoxical psoriasis. Nat Commun. 2018;9(1):25. 29295985 10.1038/s41467-017-02466-4 PMC5750213

[38] Misiak‐Galazka M , Zozula J , Rudnicka L . Palmoplantar Pustulosis: Recent Advances in Etiopathogenesis and Emerging Treatments. Am J Clin Dermatol. 2020;21(3):355‐370. 32008176 10.1007/s40257-020-00503-5 PMC7275027

[39] Bissonnette R , Suárez‐Fariñas M , Li X , et al. Based on Molecular Profiling of Gene Expression, Palmoplantar Pustulosis and Palmoplantar Pustular Psoriasis Are Highly Related Diseases that Appear to Be Distinct from Psoriasis Vulgaris. PLoS One. 2016;11(5):e0155215. 27152848 10.1371/journal.pone.0155215 PMC4859542

[40] Klemm P , Lange U . SAPHO‐Syndrom: Ein Überblick und nosologische Differenzierung von 35 Krankheitsfällen. Z Rheumatol. 2021;80(5):456‐466. 33725179 10.1007/s00393-021-00979-4 PMC8190029

[41] Kishimoto M , Taniguchi Y , Tsuji S , et al. SAPHO syndrome and pustulotic arthro‐osteitis. Mod Rheumatol. 2022;32(4):665‐674. 34967407 10.1093/mr/roab103

[42] Sonozaki H , Kawashima M , Hongo O , et al. Incidence of arthro‐osteitis in patients with pustulosis palmaris et plantaris. Ann Rheum Dis. 1981;40(6):554‐557. 6800310 10.1136/ard.40.6.554 PMC1000828

[43] Nagel T , Eger G , Kalden JR , Manger B . Arthroosteitis pustulosa, spondarthritis hyperostotica pustulo‐psoriatica, SAPHO syndrome: clinical experiences and review of the literature. Z Rheumatol. 1993;52(6):390‐397. 8147133

[44] Tsuji S , Okubo Y , Kishimoto M , et al. Modified pustulotic arthro‐osteitis diagnostic guidance 2022 ‐ Modified Sonozaki criteria ‐ Secondary publication. Mod Rheumatol. 2024;34(5):1076‐1078. 38300513 10.1093/mr/roae003

[45] Chamot AM , Benhamou CL , Kahn MF , et al. Le syndrome acné pustulose hyperostose ostéite (SAPHO). Résultats d'une enquête nationale. 85 observations. Rev Rhum Mal Osteoartic. 1987;54(3):187‐196. 2954204

[46] Hayem G . SAPHO syndrome. Rev Prat. 2004;54(15):1635‐1636. 15605575

[47] Sevrain M , Richard M‐A , Barnetche T , et al. Treatment for palmoplantar pustular psoriasis: systematic literature review, evidence‐based recommendations and expert opinion. J Eur Acad Dermatol Venereol. 2014;28 Suppl 5(s5):13‐16. 24985558 10.1111/jdv.12561

[48] Kim M , Kim M , Kim J‐W , et al. Clinical characteristics of pustulotic arthro‐osteitis in Korea. J Dermatol. 2022;49(8):762‐768. 35510638 10.1111/1346-8138.16413

[49] Veale DJ , Fearon U . The pathogenesis of psoriatic arthritis. Lancet. 2018;391(10136):2273‐2284. 29893226 10.1016/S0140-6736(18)30830-4

[50] Taylor W , Gladman D , Helliwell P , et al. Classification criteria for psoriatic arthritis: development of new criteria from a large international study. Arthritis Rheum. 2006;54(8):2665‐2673. 16871531 10.1002/art.21972

[51] Heidemeyer K , May Lee M , Cazzaniga S , et al. Palmoplantar Pustulosis: A Systematic Review of Risk Factors and Therapies. Psoriasis (Auckland, N.Z.). 2023;13:33‐58. 37772169 10.2147/PTT.S400402 PMC10522454

[52] Kim DH , Lee JY , Cho SI , Jo SJ . Risks of Comorbidities in Patients With Palmoplantar Pustulosis vs Patients With Psoriasis Vulgaris or Pompholyx in Korea. JAMA Dermatol. 2022;158(6):650‐660. 35476054 10.1001/jamadermatol.2022.1081 PMC9047771

[53] Hagforsen E , Michaëlsson K , Lundgren E , et al. Women with palmoplantar pustulosis have disturbed calcium homeostasis and a high prevalence of diabetes mellitus and psychiatric disorders: a case‐control study. Acta Derm Venereol. 2005;85(3):225‐232. 16040407 10.1080/00015550510026587

[54] Becher G , Jamieson L , Leman J . Palmoplantar pustulosis–a retrospective review of comorbid conditions. J Eur Acad Dermatol Venereol. 2015;29(9):1854‐1856. 24813766 10.1111/jdv.12545

[55] Ghoreschi K , Balato A , Enerbäck C , Sabat R . Therapeutics targeting the IL‐23 and IL‐17 pathway in psoriasis. Lancet. 2021;397(10275):754‐766. 33515492 10.1016/S0140-6736(21)00184-7

[56] Hüffmeier U , Klima J , Hayatu MD . Genetic underpinnings of the psoriatic spectrum. Med Genet. 2023;35(1):46‐54. 38835412 10.1515/medgen-2023-2005 PMC10842586

[57] Hernandez‐Cordero A , Thomas L , Smail A , et al. A genome‐wide meta‐analysis of palmoplantar pustulosis implicates TH2 responses and cigarette smoking in disease pathogenesis. J Allergy Clin Immunol. 2024;154(3):657‐665. e9. 38815935 10.1016/j.jaci.2024.05.015

[58] Yoon SY , Park HS , Lee JH , Cho S . Histological differentiation between palmoplantar pustulosis and pompholyx. J Eur Acad Dermatol Venereol. 2013;27(7):889‐893. 22691103 10.1111/j.1468-3083.2012.04602.x

[59] Wolk K , Frambach Y , Jacobi A , et al. Increased levels of lipocalin 2 in palmoplantar pustular psoriasis. J Dermatol Sci. 2018;90(1):68‐74. 29395573 10.1016/j.jdermsci.2017.12.018

[60] Brembach T‐C , Sabat R , Witte K , et al. Molecular and functional changes in neutrophilic granulocytes induced by nicotine: a systematic review and critical evaluation. Front Immunol. 2023;14:1281685. 38077313 10.3389/fimmu.2023.1281685 PMC10702484

[61] Wolk K , Wilsmann‐Theis D , Witte K , et al. Interleukin‐19 Levels Are Increased in Palmoplantar Pustulosis and Reduced following Apremilast Treatment. Int J Mol Sci. 2023;24(2):1276. 36674793 10.3390/ijms24021276 PMC9862858

[62] Liu C , Liu X , Xin H , Li X . Associations of inflammatory cytokines with palmoplantar pustulosis: a bidirectional Mendelian randomization study. Front Med (Lausanne). 2024;11:1387210. 38882664 10.3389/fmed.2024.1387210 PMC11176421

[63] Bissonnette R , Nigen S , Langley RG , et al. Increased expression of IL‐17A and limited involvement of IL‐23 in patients with palmo‐plantar (PP) pustular psoriasis or PP pustulosis; results from a randomised controlled trial. J Eur Acad Dermatol Venereol. 2014;28(10):1298‐1305. 24112799 10.1111/jdv.12272

[64] Murakami M , Hagforsen E , Morhenn V , et al. Patients with palmoplantar pustulosis have increased IL‐17 and IL‐22 levels both in the lesion and serum. Exp Dermatol. 2011;20(10):845‐847. 21732985 10.1111/j.1600-0625.2011.01325.x

[65] Sabat R , Gudjonsson JE , Brembilla NC , et al. Biology of Interleukin‐17 and Novel Therapies for Hidradenitis Suppurativa. J Interferon Cytokine Res. 2023;43(12):544‐556. 37824200 10.1089/jir.2023.0105

[66] McCluskey D , Benzian‐Olsson N , Mahil SK , et al. Single‐cell analysis implicates TH17‐to‐TH2 cell plasticity in the pathogenesis of palmoplantar pustulosis. J Allergy Clin Immunol. 2022;150(4):882‐893. 35568077 10.1016/j.jaci.2022.04.027

[67] Wolk K , Wenzel J , Tsaousi A , et al. Lipocalin‐2 is expressed by activated granulocytes and keratinocytes in affected skin and reflects disease activity in acne inversa/hidradenitis suppurativa. Br J Dermatol. 2017;177(5):1385‐1393. 28256718 10.1111/bjd.15424

[68] Beltraminelli H , Blum R . Psoriasiforme entzündliche Dermatosen. In: Cerroni L , Garbe C , Metze D , Kutzner H , Kerl H , eds. Histopathologie der Haut. Berlin, Heidelberg: Springer Berlin Heidelberg; 2015:1‐23.

[69] Balan R , Grigoraş A , Popovici D , Amălinei C . The histopathological landscape of the major psoriasiform dermatoses. Arch Clin Cases. 2019;6(3):59‐68. 34754910 10.22551/2019.24.0603.10155 PMC8565680

[70] Zaaroura H , Bergman R . How useful is periodic acid‐Schiff stain to detect fungi in biopsies from dermatoses of the palms and soles? J Cutan Pathol. 2019;46(6):418‐420. 30843246 10.1111/cup.13451

[71] Misiak‐Galazka M , Wolska H , Rudnicka L . What do we know about palmoplantar pustulosis? J Eur Acad Dermatol Venereol. 2017;31(1):38‐44. 27521275 10.1111/jdv.13846

[72] Nast A , Altenburg A , Augustin M , et al. Deutsche S3‐Leitlinie zur Therapie der Psoriasis vulgaris: Adaptiert von EuroGuiDerm ‐ Teil 1 und 2: Therapieziele und Therapieempfehlungen. J Dtsch Dermatol Ges. 2021. https://register.awmf.org/assets/guidelines/013‐001l_S3_Therapie‐Psoriasis‐vulgaris_2024‐04.pdf. [Last accessed January 15, 2025]. 10.1111/ddg.14508_g 34139080

[73] Lu JD , Lytvyn Y , Mufti A , et al. Biologic therapies associated with development of palmoplantar pustulosis and palmoplantar pustular psoriasis: a systematic review. Int J Dermatol. 2022;62(1):12‐21. 35128653 10.1111/ijd.16064

[74] Kragballe K , Larsen FG . A hydrocolloid occlusive dressing plus triamcinolone acetonide cream is superior to clobetasol cream in palmo‐plantar pustulosis. Acta Derm Venereol. 1991;71(6):540‐542. 1685841

[75] Volden G . Successful treatment of chronic skin diseases with clobetasol propionate and a hydrocolloid occlusive dressing. Acta Derm Venereol. 1992;72(1):69‐71. 1350154

[76] Umezawa Y , Nakagawa H , Tamaki K . Phase III clinical study of maxacalcitol ointment in patients with palmoplantar pustulosis: A randomized, double‐blind, placebo‐controlled trial. J Dermatol. 2016;43(3):288‐293. 26282062 10.1111/1346-8138.13064

[77] Muro M , Kawakami H , Matsumoto Y , et al. Topical combination therapy with vitamin D3 and corticosteroid ointment for palmoplantar pustulosis: A prospective, randomized, left‐right comparison study. J Dermatolog Treat. 2016;27(1):51‐53. 26108445 10.3109/09546634.2015.1052036

[78] Torres T , Galván J , Crutchley N , et al. Calcipotriol and Betamethasone Dipropionate Cream Based on PAD Technology for the Treatment of Plaque Psoriasis: A Narrative Review. Dermatol Ther (Heidelb). 2023;13(10):2153‐2169. 37740858 10.1007/s13555-023-01003-0 PMC10539254

[79] Wohlrab J , Eichner A . Supersaturation as a Galenic Concept for Improving the Cutaneous Bioavailability of Drugs in Topical Therapy. Dermatol Ther (Heidelb). 2023;13(2):391‐398. 36542293 10.1007/s13555-022-00873-0 PMC9884713

[80] Cramer N , Wellmann P , Schön MP , et al. Successful treatment of palmoplantar pustulosis with topical ruxolitinib: (in production). J Dtsch Dermatol Ges. 2025;23(12):1591‐1593. 40847898 10.1111/ddg.15854 PMC12697319

[81] S1‐Leitlinie zur UV‐Phototherapie und Photochemotherapie: Leitlinie der Deutschen Dermatologischen Gesellschaft. https://register.awmf.org/assets/guidelines/013‐029l_S1_UV‐Phototherapie__Photochemotherapie_2015‐08‐abgelaufen.pdf. [Last accessed January 15, 2025]. 10.1111/ddg.12912_g 27509439

[82] Kawada A , Matsuda H , Oiso N . Efficacy and safety of targeted narrowband ultraviolet B therapy using a flat‐type fluorescent lamp for the treatment of palmoplantar pustulosis. J Dermatol. 2013;40(9):754‐755. 23855773 10.1111/1346-8138.12228

[83] Fachinformationen Meladinine Lösungskonzentrat 0,3 %. https://medikamio.com/downloads/de‐de/drugs/meladinine‐loesungskonzentrat‐03.pdf. [Last accessed January 15, 2025].

[84] Su L , Ren J , Cheng S , et al. UVA1 vs. narrowband UVB phototherapy in the treatment of palmoplantar pustulosis: a pilot randomized controlled study. Lasers Med Sci. 2017;32(8):1819‐1823. 28699044 10.1007/s10103-017-2280-0

[85] Layton AM , Sheehan‐Dare R , Cunlife WJ . A double‐blind, placebo‐controlled trial of topical PUVA in persistent palmoplantar pustulosis. Br J Dermatol. 1991;124(6):581‐584. 2064943 10.1111/j.1365-2133.1991.tb04955.x

[86] Rosén K , Mobacken H , Swanbeck G . PUVA, etretinate, and PUVA‐etretinate therapy for pustulosis palmoplantaris. A placebo‐controlled comparative trial. Arch Dermatol. 1987;123(7):885‐889. 3300565 10.1001/archderm.1987.01660310053013

[87] Peng C , Hu Y , Chen W , et al. A randomized prospective study of different dose regimens using the 308‐nm excimer laser in the treatment of palmoplantar pustulosis. Dermatol Ther. 2021;34(5):e15079. 34333826 10.1111/dth.15079

[88] Furuhashi T , Torii K , Kato H , et al. Efficacy of excimer light therapy (308 nm) for palmoplantar pustulosis with the induction of circulating regulatory T cells. Exp Dermatol. 2011;20(9):768‐770. 21672034 10.1111/j.1600-0625.2011.01316.x

[89] Fumimori T , Tsuruta D , Kawakami T , et al. Effect of monochromatic excimer light on palmoplantar pustulosis: a clinical study performed in a private clinic by a dermatological specialist. J Dermatol. 2013;40(12):1004‐1007. 24303875 10.1111/1346-8138.12302

[90] Iga N , Otsuka A , Tanioka M , et al. Improvement of Anti‐TNF‐α Antibody‐Induced Palmoplantar Pustular Psoriasis Using a 308‐nm Excimer Light. Case Rep Dermatol. 2012;4(3):261‐264. 23275771 10.1159/000345468 PMC3531932

[91] Gianfaldoni S , Tchernev G , Wollina U , Lotti T . Pustular Palmoplantar Psoriasis Successfully Treated with Nb‐UVB Monochromatic Excimer Light: A Case‐Report. Open Access Maced J Med Sci. 2017;5(4):462‐466. 28785333 10.3889/oamjms.2017.080 PMC5535658

[92] Fachinformationen Acicutan® 10 mg/25 mg Hartkapseln. https://fachinfo.de/fi/detail/014128/acicutan‐r‐10‐mg‐25‐mg‐hartkapseln. [Last accessed January 15, 2025].

[93] Lassus A , Geiger JM . Acitretin and etretinate in the treatment of palmoplantar pustulosis: a double‐blind comparative trial. Br J Dermatol. 1988;119(6):755‐759. 2974306 10.1111/j.1365-2133.1988.tb03499.x

[94] Ettler K , Richards B . Acitretin therapy for palmoplantar pustulosis combined with UVA and topical 8‐MOP. Int J Dermatol. 2001;40(8):541‐542. 11703532 10.1046/j.1365-4362.2001.01094-3.x

[95] Reich K , Graff O , Mehta N . Oral alitretinoin treatment in patients with palmoplantar pustulosis inadequately responding to standard topical treatment: a randomized phase II study. Br J Dermatol. 2016;174(6):1277‐1281. 26800106 10.1111/bjd.14401

[96] Adişen E , Tekin O , Gülekon A , Gürer MA . A retrospective analysis of treatment responses of palmoplantar psoriasis in 114 patients. J Eur Acad Dermatol Venereol 2009;23(7):814‐819. 19470063 10.1111/j.1468-3083.2009.03197.x

[97] Kromer C , Wilsmann‐Theis D , Gerdes S , et al. Drug survival and reasons for drug discontinuation in palmoplantar pustulosis: a retrospective multicenter study. J Dtsch Dermatol Ges. 2019;17(5):503‐516. 10.1111/ddg.13834 PMC6850581 30994260

[98] Reitamo S , Erkko P , Remitz A , et al. Cyclosporine in the treatment of palmoplantar pustulosis. A randomized, double‐blind, placebo‐controlled study. Arch Dermatol. 1993;129(10):1273‐1279. 8215491

[99] Erkko P , Granlund H , Remitz A , et al. Double‐blind placebo‐controlled study of long‐term low‐dose cyclosporin in the treatment of palmoplantar pustulosis. Br J Dermatol. 1998;139(6):997‐1004. 9990362 10.1046/j.1365-2133.1998.02555.x

[100] Jin X‐H , Chen X , Mou Y , Xia J‐X . Effects of Cyclosporine on Palmoplantar Pustulosis and Serum Expression of IL‐17, IL‐23, and TNF‐α. Dermatol Ther (Heidelb). 2019;9(3):547‐552. 31240637 10.1007/s13555-019-0308-z PMC6704219

[101] Fachinformationen Ciclosporin‐1A‐Pharma. http://www.1a‐files.de/pdf/fi/2016_06_ciclosporin_1_a_kps_fi.pdf. [Last accessed January 15, 2025].

[102] Ständer H , Stadelmann A , Luger T , Traupe H . Efficacy of fumaric acid ester monotherapy in psoriasis pustulosa palmoplantaris. Br J Dermatol. 2003;149(1):220‐222. 12890235 10.1046/j.1365-2133.2003.05424.x

[103] Wilsmann‐Theis D , Kromer C , Gerdes S , et al. A multicentre open‐label study of apremilast in palmoplantar pustulosis (APLANTUS). J Eur Acad Dermatol Venereol. 2021;35(10):2045‐2050. 34077577 10.1111/jdv.17441

[104] Terui T , Okubo Y , Kobayashi S , et al. Efficacy and Safety of Apremilast for the Treatment of Japanese Patients with Palmoplantar Pustulosis: Results from a Phase 2, Randomized, Placebo‐Controlled Study. Am J Clin Dermatol. 2023;24(5):837‐847. 37233897 10.1007/s40257-023-00788-2 PMC10213585

[105] Kato N , Takama H , Ando Y , et al. Immediate response to apremilast in patients with palmoplantar pustulosis: a retrospective pilot study. Int J Dermatol. 2021;60(5):570‐578. 33454961 10.1111/ijd.15382 PMC8248100

[106] Ständer S , Syring F , Ludwig RJ , Thaçi D . Successful Treatment of Refractory Palmoplantar Pustular Psoriasis With Apremilast: A Case Series. Front Med (Lausanne). 2020;7:543944. 33178709 10.3389/fmed.2020.543944 PMC7593234

[107] KT S , Thakur V , Narang T , et al. Apremilast in treatment of palmoplantar pustulosis ‐ a case series. Int J Dermatol. 2021;60(6):e247‐e248. 33475149 10.1111/ijd.15398

[108] Marovt M , Marko PB . Apremilast monotherapy for palmoplantar pustulosis: Report of three cases. SAGE Open Med Case Rep. 2021;9:1‐3, 2050313X211034926. 10.1177/2050313X211034926 PMC8361521 34394938

[109] Frischhut N , Ratzinger G . Apremilast in the treatment of palmoplantar pustulosis : A case series. Hautarzt. 2021;72(3):252‐256. 32876701 10.1007/s00105-020-04676-2 PMC7935730

[110] Mössner R , Hoff P , Mohr J , Wilsmann‐Theis D . Successful therapy of palmoplantar pustulosis with tofacitinib‐Report on three cases. Dermatol Ther. 2020;33(4):e13753. 32495413 10.1111/dth.13753

[111] Haynes D , Topham C , Hagstrom E , Greiling T . Tofacitinib for the treatment of recalcitrant palmoplantar pustulosis: A case report. Australas J Dermatol. 2020;61(1):e108‐e110. 31318041 10.1111/ajd.13117

[112] Koga T , Sato T , Umeda M , et al. Successful treatment of palmoplantar pustulosis with rheumatoid arthritis, with tofacitinib: Impact of this JAK inhibitor on T‐cell differentiation. Clin Immunol. 2016;173:147‐148. 27720846 10.1016/j.clim.2016.10.003

[113] Mohr J , Rahbar Kooybaran N , Schön MP , Mössner R . Response of palmoplantar pustulosis to upadacitinib. J Dtsch Dermatol Ges. 2023;21(3):280‐282. 10.1111/ddg.14969 36730648

[114] Gleeson D , Barker JNWN , Capon F , et al. Are Janus kinase inhibitors an effective treatment for palmoplantar pustulosis? A critically appraised topic. Br J Dermatol. 2023;188(4):471‐473. 36715624 10.1093/bjd/ljac130

[115] Du N , Yang J , Zhang Y , et al. Successful treatment of refractory palmoplantar pustulosis by upadacitinib: report of 28 patients. Front Med (Lausanne). 2024;11:1476793. 39568752 10.3389/fmed.2024.1476793 PMC11576273

[116] Liu S , Yu Y , Liu Y , et al. Efficacy of baricitinib in synovitis, acne, pustulosis, hyperostosis, and osteitis syndrome: A case series. Joint Bone Spine. 2023;90(5):105587. 37127258 10.1016/j.jbspin.2023.105587

[117] Rahbar Kooybaran N , Tsianakas A , Assaf K , et al. Response of palmoplantar pustulosis to upadacitinib: A case series of five patients. J Dtsch Dermatol Ges. 2023;21(11):1387‐1392. 10.1111/ddg.15176 37605445

[118] Wohlrab J , Kegel T , Große R , Eichner A . Recommendations for risk minimization when using Janus kinase inhibitors for the treatment of chronic inflammatory skin diseases. J Dtsch Dermatol Ges. 2023;21(8):845‐851. 10.1111/ddg.15136 37345890

[119] Bissonnette R , Poulin Y , Bolduc C , et al. Etanercept in the treatment of palmoplantar pustulosis. J Drugs Dermatol. 2008;7(10):940‐946. 19112757

[120] Wu J , Smogorzewski J . Ustekinumab for the treatment of paradoxical skin reactions and cutaneous manifestations of inflammatory bowel diseases. Dermatol Ther. 2021;34(3):e14883. 33594811 10.1111/dth.14883

[121] Au S‐C , Goldminz AM , Kim N , et al. Investigator‐initiated, open‐label trial of ustekinumab for the treatment of moderate‐to‐severe palmoplantar psoriasis. J Dermatolog Treat. 2013;24(3):179‐187. 22390688 10.3109/09546634.2012.672710

[122] Fachinformationen Tremfya® 100 mg Injektionslösung. https://www.fachinfo.de/fi/detail/021847/tremfya‐r‐100‐mg‐injektionsloesung.

[123] Zheng R , Ito YM , Yunoki M , et al. Design and implementation of an adaptive confirmatory trial in Japanese patients with palmoplantar pustulosis. Contemp Clin Trials Commun. 2022;28:100935. 35711679 10.1016/j.conctc.2022.100935 PMC9192787

[124] Yamanaka K , Okubo Y , Yasuda I , et al. Efficacy and safety of risankizumab in Japanese patients with generalized pustular psoriasis or erythrodermic psoriasis: Primary analysis and 180‐week follow‐up results from the phase 3, multicenter IMMspire study. J Dermatol. 2023;50(2):195‐202. 36514850 10.1111/1346-8138.16667 PMC10107196

[125] Terui T , Kobayashi S , Okubo Y , et al. Efficacy and Safety of Guselkumab, an Anti‐interleukin 23 Monoclonal Antibody, for Palmoplantar Pustulosis: A Randomized Clinical Trial. JAMA Dermatol. 2018;154(3):309‐316. 29417135 10.1001/jamadermatol.2017.5937 PMC5885837

[126] Terui T , Kobayashi S , Okubo Y , et al. Efficacy and Safety of Guselkumab in Japanese Patients With Palmoplantar Pustulosis: A Phase 3 Randomized Clinical Trial. JAMA Dermatol. 2019;155(10):1153‐1161. 31268476 10.1001/jamadermatol.2019.1394 PMC6613288

[127] Wilsmann‐Theis D , Patt S , Pinter A , et al. Efficacy and safety of guselkumab in European patients with palmoplantar pustulosis: A multi‐center, single‐arm clinical trial (GAP study). JAAD Int. 2025;18:69‐78. 39618912 10.1016/j.jdin.2024.09.001 PMC11607598

[128] Okubo Y , Murakami M , Kobayashi S , et al. Risankizumab in Japanese patients with moderate‐to‐severe palmoplantar pustulosis: Results from the randomized, phase 3 JumPPP study. J Dermatol. 2025;52(4):593‐602. 40001318 10.1111/1346-8138.17659 PMC11975171

[129] Poortinga S , Balakirski G , Kromer C , et al. The challenge of palmoplantar pustulosis therapy: Are Interleukin‐23 inhibitors an option? J Eur Acad Dermatol Venereol. 2021;35(12):e907‐e911. 34309915 10.1111/jdv.17560

[130] Mrowietz U , Bachelez H , Burden AD , et al. Secukinumab for moderate‐to‐severe palmoplantar pustular psoriasis: Results of the 2PRECISE study. J Am Acad Dermatol. 2019;80(5):1344‐1352. 30716404 10.1016/j.jaad.2019.01.066

[131] Mrowietz U , Bachelez H , Burden AD , et al. Efficacy and safety of secukinumab in moderate to severe palmoplantar pustular psoriasis over 148 weeks: Extension of the 2PRECISE study. J Am Acad Dermatol. 2021;84(2):552‐554. 32565211 10.1016/j.jaad.2020.06.038

[132] Reolid A , Armesto S , Sahuquillo‐Torralba A , et al. Secukinumab is effective in the treatment of recalcitrant palmoplantar psoriasis and palmoplantar pustular psoriasis in a daily practice setting. J Am Acad Dermatol. 2022;87(3):705‐709. 35640798 10.1016/j.jaad.2022.05.047

[133] Okubo Y , Kobayashi S , Murakami M , et al. Efficacy and Safety of Brodalumab, an Anti‐interleukin‐17 Receptor A Monoclonal Antibody, for Palmoplantar Pustulosis: 16‐Week Results of a Randomized Clinical Trial. Am J Clin Dermatol. 2024. 10.1007/s40257-024-00876-x PMC11358179 38954226

[134] Passeron T , Perrot J‐L , Jullien D , et al. Treatment of Severe Palmoplantar Pustular Psoriasis With Bimekizumab. JAMA Dermatol. 2024;160(2):199‐203. 38054800 10.1001/jamadermatol.2023.5051 PMC10701662

[135] Xia R , Liu J , Gao Y , et al. Local injection of micro‐dose anti‐interleukin‐17A antibody for palmoplantar pustulosis: A real‐world study. Clin Immunol. 2023;253:109694. 37433424 10.1016/j.clim.2023.109694

[136] Mrowietz U , Burden AD , Pinter A , et al. Spesolimab, an Anti‐Interleukin‐36 Receptor Antibody, in Patients with Palmoplantar Pustulosis: Results of a Phase IIa, Multicenter, Double‐Blind, Randomized, Placebo‐Controlled Pilot Study. Dermatol Ther (Heidelb). 2021;11(2):571‐585. 33661508 10.1007/s13555-021-00504-0 PMC8019016

[137] Cro S , Cornelius VR , Pink AE , et al. Anakinra for palmoplantar pustulosis: results from a randomized, double‐blind, multicentre, two‐staged, adaptive placebo‐controlled trial (APRICOT)*. Br J Dermatol. 2021;186(2):245‐256. 34411292 10.1111/bjd.20653 PMC9255857

[138] Tauber M , Viguier M , Alimova E , et al. Partial clinical response to anakinra in severe palmoplantar pustular psoriasis. Br J Dermatol. 2014;171(3):646‐649. 24684162 10.1111/bjd.13012

[139] Zheng Y‐X , Chen X‐B , Wang Z‐Y , et al. Efficacy of dupilumab in palmoplantar pustulosis treatment highlights the role of Th2 inflammation. Allergy. 2024;79(5):1361‐1364. 38193274 10.1111/all.16019

[140] Efficacy and Tolerability of Deucravacitinib in the Management of Palmoplantar Pustulosis: US NCT number NCT07000630. https://trial.medpath.com/clinical‐trial/a4603fd10559889c/deucravacitinib‐palmoplantar‐pustulosis‐trial. Updated 2025‐25.

[141] A Phase 2, Randomized, Placebo‐controlled, Double‐blind, Multiple Dose Study to Evaluate the Efficacy and Safety of ANB019 in Subjects with Palmoplantar Pustulosis: US NCT number NCT03633396. https://www.clinicaltrialsregister.eu/ctr‐search/trial/2017‐004022‐15/results. Updated May 21, 2022.

[142] Mrowietz U , van de Kerkhof PCM . Management of palmoplantar pustulosis: do we need to change? Br J Dermatol. 2011;164(5):942‐946. 21275942 10.1111/j.1365-2133.2011.10233.x

[143] Kim SR , Choi Y‐G , Jo SJ . Effect of smoking cessation on psoriasis vulgaris, palmoplantar pustulosis and generalized pustular psoriasis. Br J Dermatol. 2024;191(2):225‐232. Klicken oder tippen Sie hier, um Text einzugeben. 38534203 10.1093/bjd/ljae130

